# Bis-BODIPY linked-triazole based on catechol core for selective dual detection of Ag^+^ and Hg^2+^†

**DOI:** 10.1039/d0ra09686e

**Published:** 2021-01-19

**Authors:** Worakrit Saiyasombat, Supavadee Kiatisevi

**Affiliations:** Department of Chemistry, Center of Excellence for Innovation in Chemistry (PERCH-CIC), Faculty of Science, Mahidol University Rama VI Rd, Rajthevi Bangkok 10400 Thailand supavadee.mon@mahidol.edu +66-2-354-7151 +66-2-201-5150; Center of Sustainable Energy and Green Materials, Faculty of Science, Mahidol University Salaya Putthamonthon Nakhon Pathom 73170 Thailand

## Abstract

Herein, we introduced a new chemosensor, bis-BODIPY linked-triazole based on catechol (BODIPY-OO) prepared by bridging two units of BODIPY fluorophore/triazole binding group with a catechol unit. A solution of this compound displayed 4- and 2-fold enhancements in fluorescence intensity after adding a mole equivalent amount of Ag^+^ and Hg^2+^ ions in methanol media, respectively. ^1^H NMR titrations of BODIPY-OO with Ag^+^ and Hg^2+^ suggested that the triazole was involved in the recognition process. BODIPY-OO showed high sensitivity toward Ag^+^ and Hg^2+^ over other metal ions with detection limits of 0.45 μM and 1 μM, respectively. It can also distinguish Hg^2+^ from Ag^+^ by addition of an EDTA. This compound can therefore be employed as practical fluorescent probe for monitoring the presence of Ag^+^ and Hg^2+^ ions.

## Abstract 2

BODIPY–triazole–catechol combination serves as a “turn-on” fluorescent probe for dual detection and differentiation of Hg^2+^ and Ag^+^ ions.

## Graphical abstract

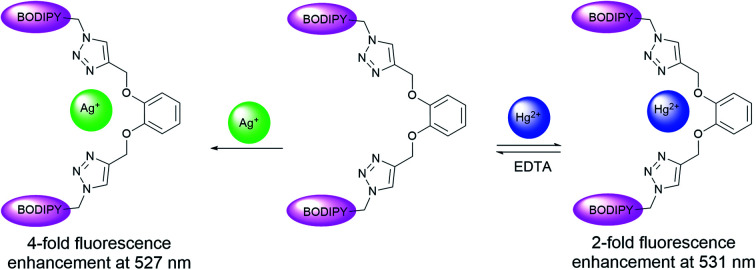

## Introduction

Since toxicity of heavy and transition metal ions affects biological and environmental systems, it is highly desirable to develop sensing probes to monitor and quantify these ions.^1–3^ Specifically, mercury is used in various industries such as pharmaceuticals, batteries, semi-conductors, agriculture, and paper industry.^4^ It is also widely used in dentistry and scientific research as amalgam.^5,6^ The Agency for Toxic Substances and Disease Registry (ATSDR) has ranked mercury as the third of most toxic substances.^7^ Its widespread use leads to the release and accumulation of mercury in general environment.^8^ Humans are exposed to mercury mainly through consumption of contaminated food, inhalation, and even through the skin causing gut lining and kidney damage, pneumonitis and disorder of many parts of the brain and peripheral nervous system.^6^ In addition to mercury, silver has been extensively used by many industrial applications such as in photovoltaics, photography, jewelry and silverware production.^9,10^ Silver has also long been known for its antimicrobial properties and has been used for years in the medical field.^11^ Although it shows a low level of toxicity to human,^12^ high levels of the silver ion can inhibit glutathione (GSH), markers, and antioxidant enzymes.^13^ Silver also reduces superoxide dismutase (SOD) levels causing an oxidative stress and production of reactive oxygen species (ROS). In addition, prolonged exposure to silver may lead to irreversible discoloration of skin or eyes, *i.e.* argyria or argyrosis.^14^

There are a lot of analytical techniques for mercury and silver determination such as atomic absorption spectrometry (AAS),^15^ inductively coupled plasma mass spectroscopy (ICP-MS),^16^ inductively coupled plasma-atomic emission spectrometry (ICP-AES),^17^ and electrochemical methods.^18^ However, these methods are time-consuming and require sophisticated instrumentation. A simple, inexpensive, and efficient alternative is fluorescent sensor where binding of the analyte leads to a change in fluorescence which requires only cheap routine spectrometers.^19,20^ A good fluorescent sensor should not only be sensitive and selective but also allows real-time monitoring of targeted metal ions with low response time.^21,22^

Small-molecule fluorescent chemosensors are usually composed of a fluorophore and a chelating or recognition unit. The fluorophores with excellent optical properties, such as coumarin,^23^ fluorescein,^24^ rhodamine^25^ and BODIPY^26^ provide highly sensitive fluorescent chemosensors. Among these, BODIPY derivatives show great potentials owing to their outstanding photophysical properties, *i.e.* spectral bands tunable along the whole visible spectrum, high molar absorption coefficients (>80 000 cm^−1^ M^−1^), and high fluorescence quantum yields.^27,28^ Owing to their soft character, nitrogen heterocyclic systems, *e.g.* pyridine,^29^ quinoline,^30^ imidazole,^31^ and triazole^32^ are mostly used as recognition units for various metal ions and tend to react with soft acids such as mercury and silver ions. The triazole ring has emerged as an exciting moiety in the study of sensors. This stems from a robust and green protocol for the synthesis of triazole derivatives.^33^ In addition to metal sensors, triazole is extensively used in the synthesis of catalyst,^34^ dendrimers,^35^ biomolecule conjugates,^36^ and metal organic frameworks.^37^

**Scheme 1:**
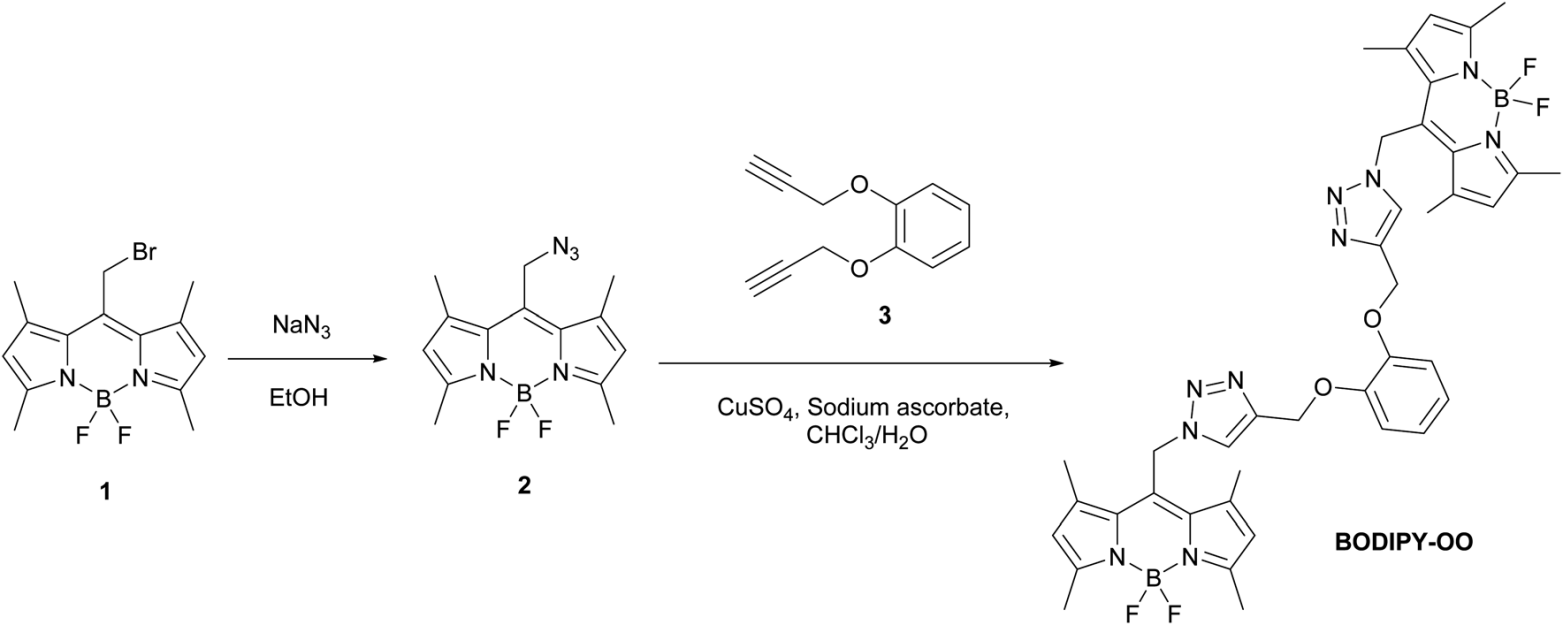
BODIPY-OO synthesis.

BODIPY–triazole combinations were reported as fluorescent chemosensors for the detection of metal ions, *e.g.* Hg^2+^,^38^ Cu^2+^,^39^ Al^3+^,^40^ and Ag^+^.^41^ This construction pattern offers following advantages: (i) click reaction affords triazoles in high yields with simple synthetic protocols; (ii) two triazole units acting as a chelation pocket can efficiently capture metal ions; (iii) the presence of two BODIPY fluorophore units improves the sensitivity of detection; and (iv) optical properties of the triazole containing BODIPY are easily tuned by modifications to spacers and chelating moieties.^38–41^ However, there are no reports on using such a fluorescent chemosensor template for recognizing multiple target ions which has the advantages of being cost-effective and highly efficient. In addition, the fluorescent chemosensors that can serve as bifunctional detection of mercury and silver ion are still rare.^42–45^ Herein, we designed and synthesized a new fluorescent sensor (BODIPY-OO) composed of two units of azido-BODIPY (2) and one unit of 1,2-bis(prop-2-ynyloxy)benzene (3) covalently linked by an alkyne–azide click chemistry approach (Scheme 1). Its spectroscopic properties were investigated. BODIPY-OO presented “turn-on” fluorescence behaviors toward the silver and mercury ions over other interfering metal ions.

## Experimental

### Materials and equipment

Unless otherwise noted, all chemicals are of analytical reagent grade obtained from commercial suppliers and are used without further purification. Dichloromethane (CH_2_Cl_2_) was distilled from calcium hydride and stored under 4 Å molecular sieves. Water utilized in the analysis processes was obtained from Millipore system and deionized prior to use. Azido BODIPY 2 was synthesized following literature procedures.^47^ Alkyne 3 was prepared by conventional propargylation reaction using propargyl bromide and K_2_CO_3_. Column chromatography was performed on Merck silica gel 60 (70–230 mesh). ^1^H and ^13^C NMR spectra were recorded on Bruker Ascend™ 400 MHz spectrometers. The chemical shifts (*δ*) and coupling constants (*J*) are reported in parts per million (ppm) and Hertz (Hz), respectively. Tetramethylsilane (TMS) or residual non-deuterated solvents are used as internal reference materials in ^1^H NMR experiments. HRMS spectra were recorded on a HR-TOF-MS Micromass model VQ-TOF2. UV-Vis and fluorescence spectrometers were monitored on a UV-2600 (Shimadzu) and a FluoroMax® Plus (HORIBA), respectively.

### Syntheses

#### Azido-BODIPY (2)

1 (100 mg, 0.29 mmol) was dissolved in ethanol (50 mL). To this mixture, sodium azide (89.0 mg, 1.37 mmol) was added. The round-bottom flask was then wrapped with aluminum foil and placed under argon. The reaction was stirred at 40 °C for 18 h. The solvent was removed under reduced pressure. The crude mixture was dissolved in ethyl acetate, washed with water, and dried over anhydrous Na_2_SO_4_. After removal of the organic solvent, the crude product was then purified on a silica gel column using an elution of 50% CH_2_Cl_2_ in hexane to afford an orange-red solid in 80% yield (70 mg). ^1^H NMR (400 MHz, CDCl_3_): *δ* 6.10 (s, 2H), 4.60 (s, 2H), 2.53 (s, 6H), 2.46 (s, 6H). ^13^C NMR (100 MHz, CDCl_3_): *δ* 156.81, 141.36, 133.23, 132.18, 122.70, 44.78, 16.23, 14.83. HRMS (ESI) *m*/*z*: calcd for C_14_H_16_BF_2_N_5_Na [M + Na]^+^, 326.1359; found, 326.1360.

#### 1,2-Bis(prop-2-yn-1-yloxy)benzene (3)

Propargyl bromide (294 mg, 2 mmol) and K_2_CO_3_ (276 mg, 2 mmol) were added to a solution of *o*-dihydroxybenzene (100 mg, 0.91 mmol) in acetone (5 mL). The mixture was heated to refluxing under argon for 18 h. After removal of solvent, the crude product was extracted with ethyl acetate, washed with water, and dried over anhydrous Na_2_SO_4_. It was then evaporated under reduced pressure and purified by chromatography on silica gel to afford 122 mg (85%) of 3 as a pale-yellow oil. ^1^H NMR (400 MHz, CDCl_3_): *δ* 7.08–7.05 (m, 2H), 7.01–6.97 (m, 2H), 4.76 (d, *J* = 2.2 Hz, 4H), 2.51 (t, *J* = 2.2 Hz, 2H). ^13^C NMR (100 MHz, CDCl_3_): *δ* 147.79, 122.32, 115.33, 78.75, 75.87, 57.06. HRMS (ESI) *m*/*z*: calcd for C_12_H_10_NaO_2_ [M + Na]^+^, 209.0573; found, 209.0572.

#### Sensor (BODIPY-OO)

In a Schlenk tube, 3 (20 mg, 0.10 mmol) and 2 (65 mg, 0.21 mmol) were stirred in 3 mL of CHCl_3_ at room temperature under argon. To the solution mixture were added 20 mol% of CuSO_4_·5H_2_O and 40 mol% of sodium ascorbate dissolved in deionized water. The solution was stirred for 18 h, poured into water and extracted with CH_2_Cl_2_. The combined organic layers were washed with water and dried over anhydrous Na_2_SO_4_. After solvent evaporation, the crude product was further purified by column chromatography with a gradient eluent (25%, 50%, and 100% EtOAc in hexane). BODIPY-OO was obtained as a red solid in 66% yield (52 mg, 0.066 mmol). ^1^H NMR (400 MHz, CDCl_3_): *δ* 7.57 (s, 2H), 6.96–6.92 (m, 2H), 6.86–6.82 (m, 2H), 6.08 (s, 4H), 5.77 (s, 4H), 5.13 (s, 4H), 2.53 (s, 12H), 2.15 (s, 12H). ^13^C NMR (100 MHz, CDCl_3_): *δ* 157.81, 148.55, 144.49, 141.56, 132.36, 129.95, 123.16, 122.57, 122.23, 116.65, 63.56, 45.74, 15.82, 14.89. ^11^B NMR (128 MHz, CDCl_3_): *δ* −0.45 (t, *J*_BF_ = 33.3 Hz). ^19^F NMR (377 MHz, CDCl_3_): *δ* −145.9 (q, *J*_FB_ = 33.9 Hz). HRMS (ESI) *m*/*z*: calcd for C_40_H_42_B_2_F_4_N_10_NaO_2_ [M + Na]^+^, 815.3507; found, 815.3509.

### Measurements of UV-Vis and fluorescence spectra

In UV-Vis studies, all metal salts including NaNO_3_, Mg(NO_3_)_2_·6H_2_O, Fe(NO_3_)_3_·9H_2_O, Co(NO_3_)_2_·6H_2_O, Ni(NO_3_)_2_·6H_2_O, Cu(NO_3_)_2_·3H_2_O, Zn(NO_3_)_2_·6H_2_O, Cd(NO_3_)_2_·4H_2_O, AgNO_3_, Pb(NO_3_)_2_, Hg(NO_3_)_2_, and Cr(NO_3_)_3_·9H_2_O were prepared in deionized water to get 10^−2^ M stock solutions. Solutions of the BODIPY-OO sensor (5 μM) were prepared in methanol. Test solutions were prepared by mixing 30 μL of each metal stock solution with 3 mL of the sensor solution in a quartz cuvette (path length = 1 cm). In fluorescence studies, solutions of sensor (0.5 μM) were prepared in methanol. Test solutions were prepared by mixing 3 μL of the metal stock solution with 3 mL of the sensor solution in a quartz cuvette (path length = 1 cm). The excitation wavelength was set at 470 nm and the emission wavelength range was 500–700 nm. The slit width was 3.0 nm for both excitation and emission experiments.

### Fluorescence titrations

A solution (1 μM) of the BODIPY-OO sensor was prepared in methanol. 3–30 μL of a 10^−3^ M stock solution of Hg(NO_3_)_2_ or AgNO_3_ were added to 3 mL of the sensor solution. After mixing them in a quartz cuvette (path length = 1 cm) for a few seconds, fluorescence spectra were taken at room temperature.

### Competition experiments

A solution of the sensor BODIPY-OO (0.5 μM) was prepared in MeOH. Stock solutions (10^−2^ M) of various metal ions were prepared. 3 μL of each metal solution was added to 3 mL of the sensor solution to make 20 equiv. of the metal ion. 3 μL of the Hg^2+^ or Ag^+^ solution was then added into the mixed solution to get 20 equiv. of Hg^2+^ or Ag^+^. After mixing them for a few seconds, fluorescence spectra were taken at room temperature.

### ^1^H NMR titrations

A solution of the sensor BODIPY-OO (1.00 mg, 0.001 mmol) in DMSO-d_6_ was prepared. 2–6 μL of 0.32 M AgNO_3_ solution in DMSO-d_6_ was added to the sensor solution to make a 1 : 0.5, 1 : 1, and 1 : 1.5 molar ratios of ligand to metal. After shaking them for a minute, ^1^H NMR spectra were taken at room temperature. For Hg^2+^, the same procedure was performed but HgCl_2_ and acetone-d_6_ were used as mercury source and solvent, respectively.

### Computational calculation

The structure and electronic properties of BODIPY-OO, BODIPY-OO–Ag^+^ and BODIPY-OO–Hg^2+^ were calculated using density functional theory (DFT) and its extension to the time-dependent formulation (TDDFT) as implemented in Gaussian 09 package.^48^ The ground state structures were optimized in gas-phase with the B3LYP^49,50^ functional and 6-31G(d) basis set^51,52^ for the main group elements and LANL2DZ effective core potential (ECP)^53–55^ for Hg and Ag atoms. TDDFT calculations of the 10 lowest singlet–singlet transitions were carried out on the optimized ground state geometries using CAM-B3LYP^56^/6-31G(d), and CAM-B3LYP/GENECP for the free ligand and its complexes, respectively, adopting the conductor-like polarization (CPCM)^57,58^ model for the solvent (methanol) effect. Molecular orbitals were plotted using Avogadro program.^59,60^

## Results and discussion

### Construction of BODIPY-OO fluorescent platform

BODIPY-OO designed as a sensor for detection of multiple metal ions contains a catechol moiety and two triazole units in the binding core. Copper-catalyzed azide–alkyne cycloaddition (CuAAC) was used to synthesize BODIPY-OO as depicted in Scheme 1. Azido-BODIPY 2 was obtained by azidation of its bromo analog 1, while the terminal bisalkyne 3 was prepared by the propargylation reaction of catechol. The click reaction between bisalkyne-terminated catechol ether and azido-terminated BODIPY in the presence of CuSO_4_ and sodium ascorbate in CHCl_3_/H_2_O media resulted in the formation of the target compound in 66% yield.

BODIPY-OO was purified by column chromatography and characterized by ^1^H, ^13^C, ^11^B, ^19^F NMR, and FTIR spectroscopy, and HRMS spectrometry. The triazole protons of BODIPY-OO in the ^1^H NMR spectrum resonated at *δ* 7.57 ppm as a singlet. Aromatic protons of the catechol unit appeared over the range of *δ* 6.96–6.92 ppm and 6.86–6.82 ppm whereas signals at *δ* 6.08 ppm corresponded to protons at 2,6 positions of the BODIPY core. ^1^H chemical shifts of methylene protons attached to the BODIPY core and those attached to oxygen atoms were observed at 5.77 and 5.13 ppm, respectively. Methyl protons of the BODIPY units resonated at *δ* 2.53 and 2.15 ppm. In addition, the ^11^B and ^19^F NMR spectra of BODIPY-OO exhibited a typical triplet (^11^B) and quartet (^19^F) due to B–F coupling (Fig. S15 and S16, ESI†).

### Sensing behaviors of BODIPY-OO

**Fig. 1:**
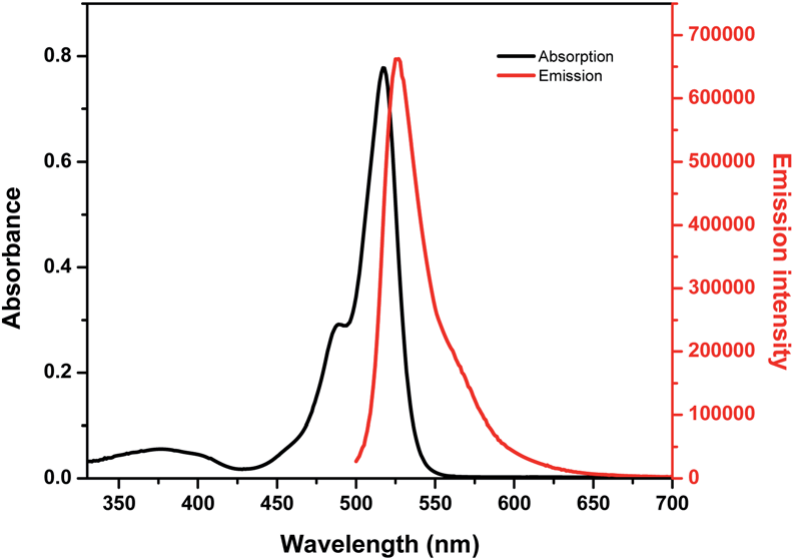
Absorption and emission spectra of BODIPY-OO (5 μM) in MeOH (*λ*_ex_ = 470 nm, slit: 3).

Optical properties of BODIPY-OO in methanol were first studied by absorption and emission spectroscopic methods (Fig. 1). The absorption spectrum displays three typical bands in the visible region: an intense main band at 517 nm attributed to typical π–π* transition (S_0_ → S_1_) of the BODIPY units; a small shoulder on the high-energy side of the main band at 489 nm ascribed to the 0–1 vibrational transition; and a weaker broad band at 375 nm which can be assigned to S_0_ → S_*n*_ (*n* ≥ 2) transitions.^61^ The fluorescence spectrum of BODIPY-OO was obtained after excitation within the spectral region of the absorption maximum which showed a single green fluorescence peak at 527 nm. BODIPY-OO has a fluorescence quantum yield (*Φ*_F_) of 0.06 relative to fluorescein (*Φ*_F_ = 0.95 in 0.1 N NaOH) as shown in Table 1.^46^

**Table 1:** Photophysical properties of BODIPY-OO and its complexes

Compound	*λ* ^max^_abs_ (nm)	*ε* (M^−1^ cm^−1^)	*λ* ^max^_em_ (nm)	*Φ*_F_ a	Binding constant (M^−1^)
BODIPY-OO	517	1.5 × 10^5^	527	0.06	—
BODIPY-OO–Ag^+^	517	1.8 × 10^5^	527	0.22	1.56 × 10^5^
BODIPY-OO–Hg^2+^	521	1.6 × 10^5^	531	0.12	1.41 × 10^5^

a Relative to fluorescein in 0.1 N NaOH (*Φ*_F_ = 0.95).^46^

**Fig. 2:**
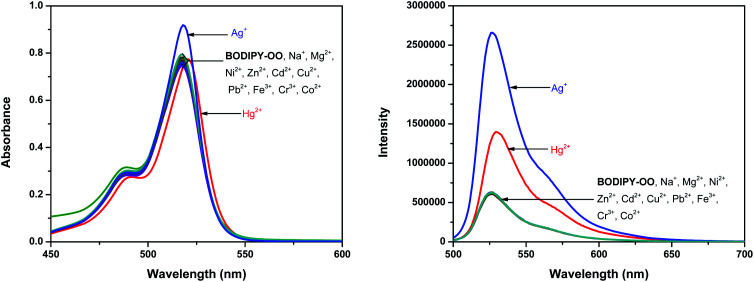
Left: absorption spectra of BODIPY-OO (5 μM) in the presence of various metal ions (20 equiv.) in MeOH. Right: emission spectra of BODIPY-OO (0.5 μM) in the presence of various metal ions (20 equiv.) in MeOH (*λ*_ex_ = 470 nm, slit: 3).

**Fig. 3:**
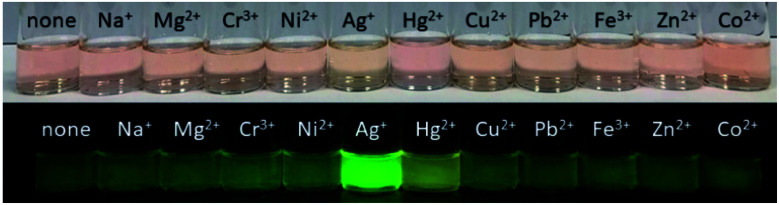
Top: photograph of BODIPY-OO (5 μM) toward tested metal ions (20 equiv.) in MeOH. Bottom: fluorogenic responses of BODIPY-OO (5 μM) toward tested metal ions (20 equiv.) in MeOH under UV light (*λ*_ex_ = 365 nm).

Our investigation began with the evaluation of the optical behaviors of BODIPY-OO in response to the addition (20 equiv.) of different ions such as Na^+^, Mg^2+^, Fe^3+^, Co^2+^, Ni^2+^, Cu^2+^, Zn^2+^, Cd^2+^, Ag^+^, Pb^2+^, Hg^2+^, and Cr^3+^. Fluorescence and UV-visible spectra indicated that BODIPY-OO was only sensitive to the Hg^2+^ and Ag^+^ ions (Fig. 2). Both ions increased the fluorescence intensity of the probe BODIPY-OO, when excited with *λ*_ex_ = 470 nm. The addition of Hg^2+^ to BODIPY-OO resulted in a small redshift of the absorption spectrum whereas the emission curves displayed a 2-fold enhancement of the fluorescence intensity at 531 nm with *Φ*_F_ of 0.12. In contrast, the addition of Ag^+^ led to a hyperchromic shift with molar extinction coefficient increasing from 1.5 × 10^5^ M^−1^ cm^−1^ (BODIPY-OO) to 1.8 × 10^5^ M^−1^ cm^−1^ (BODIPY-OO–Ag^+^). Notably, the chelation of BODIPY-OO with the Ag^+^ ion increased the fluorescence intensity at 527 nm of *ca.* 4-fold (*Φ*_F_ = 0.22) with respect to BODIPY-OO (*Φ*_F_ = 0.06). As shown in Fig. 3, our probe exhibited turn-on fluorescence toward Ag^+^ and Hg^2+^ likely as a result of chelation-enhanced fluorescence (CHEF) effect^62,63^ that is caused by the restriction of the intramolecular rotations in BODIPY-OO.^64^ The effect also decreases the non-radiative decay of the excited state. Notably, these results are different from the previously reported bifunctional probes for Ag^+^ and Hg^2+^ which mainly involved turn-off processes due to heavy atom effect and spin–orbit coupling (Table S1, ESI†).^42,65–67^

### Fluorescence titrations of BODIPY-OO with different ion concentrations

**Fig. 4:**
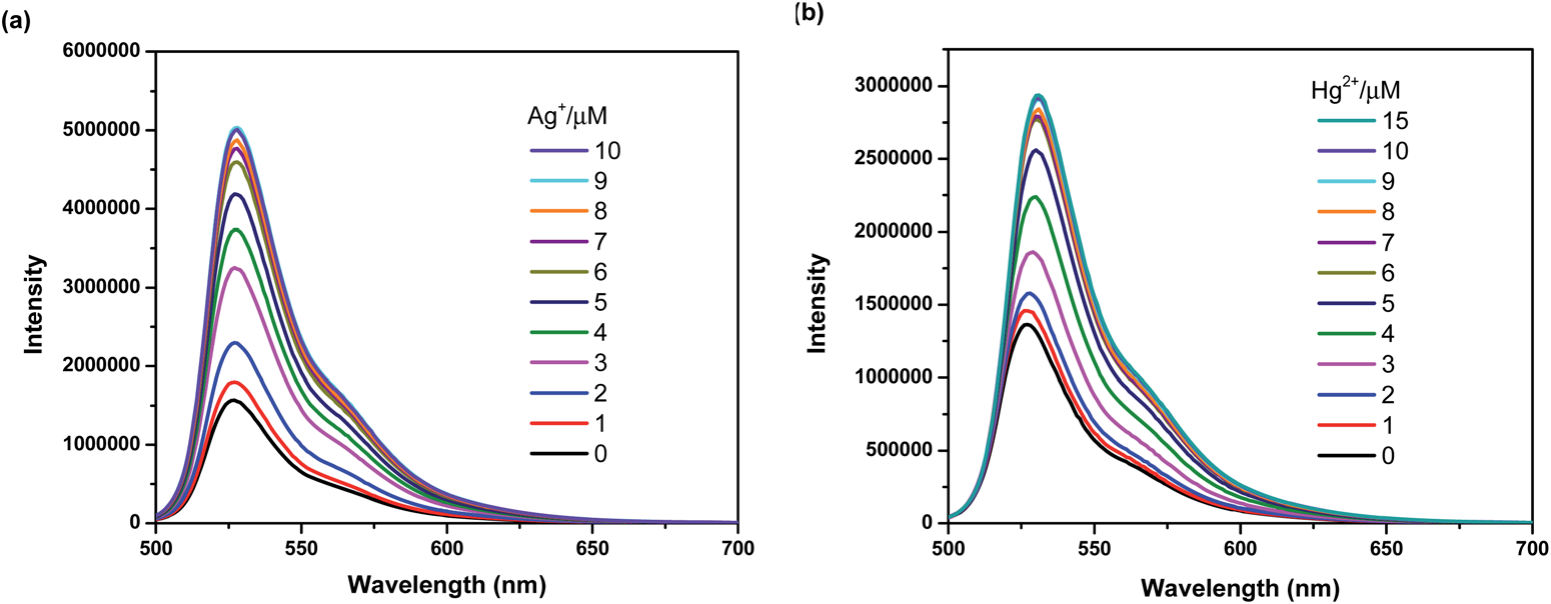
(a) Fluorescence spectra of BODIPY-OO (1 μM) with Ag^+^ in MeOH (*λ*_ex_ = 470 nm, slit: 3). (b) Fluorescence spectra of BODIPY-OO (1 μM) with Hg^2+^ in MeOH (*λ*_ex_ = 470 nm, slit: 3).

To further investigate the sensing behaviors of BODIPY-OO toward various concentrations of Ag^+^ and Hg^2+^, the fluorescence titration experiment was performed. The plots of fluorescence intensity as a function of the Ag^+^ and Hg^2+^ concentrations are shown in Fig. 4a and b, respectively. Upon sequential addition of Ag^+^ (0–10 equiv.) to the solution of BODIPY-OO, the fluorescence intensity of BODIPY-OO gradually increased and became saturated when the amount of Ag^+^ reached 9 equivalents when excited with *λ*_ex_ = 470 nm. The detection limit of BODIPY-OO for the Ag^+^ detection was estimated to be 0.45 μM using the equation: LOD = 3*σ*/*S*,^68^ where *S* is the slope of the curve between the fluorescence intensities of the probe BODIPY-OO and the concentrations of Ag^+^, and *σ* is the standard deviation of four replicate measurements of the zero level (*σ*) (Fig. S1b, ESI†). After addition of increasing concentrations of Hg^2+^ (0–15 equiv.) to the solution of BODIPY-OO, the fluorescence intensity gradually increased, and eventually reached the plateau when the amount of Hg^2+^ reached 9 equivalents. Furthermore, the emission peak gradually redshifted from 527 nm to 531 nm. According to the fluorescence titration experiment, the detection limit of BODIPY-OO for the Hg^2+^ detection was 1 μM (Fig. S2b, ESI†).

To obtain further insight into the binding characteristics of the Ag^+^ and Hg^2+^ ions with BODIPY-OO, Job plot analysis was carried out using fluorescence experiments in the presence of various molar fractions of Ag^+^ and Hg^2+^, respectively (Fig. S3 and S4, ESI†). A maximum emission was observed when the molar fraction of each metal ion reached the value of 0.5, indicating the formation of a 1 : 1 binding stoichiometry of both interactions between BODIPY-OO and Ag^+^, and between BODIPY-OO and Hg^2+^. In addition, association constant (*K*_a_) of BODIPY-OO with Ag^+^ was calculated to be 1.56 × 10^5^ M^−1^ using the Benesi–Hildebrand equation^69^ (Fig. S5, ESI†) and *K*_a_ of BODIPY-OO with Hg^2+^ was calculated to be 1.41 × 10^5^ M^−1^ (Fig. S6, ESI†).

### Interference of other metal ions

**Fig. 5:**
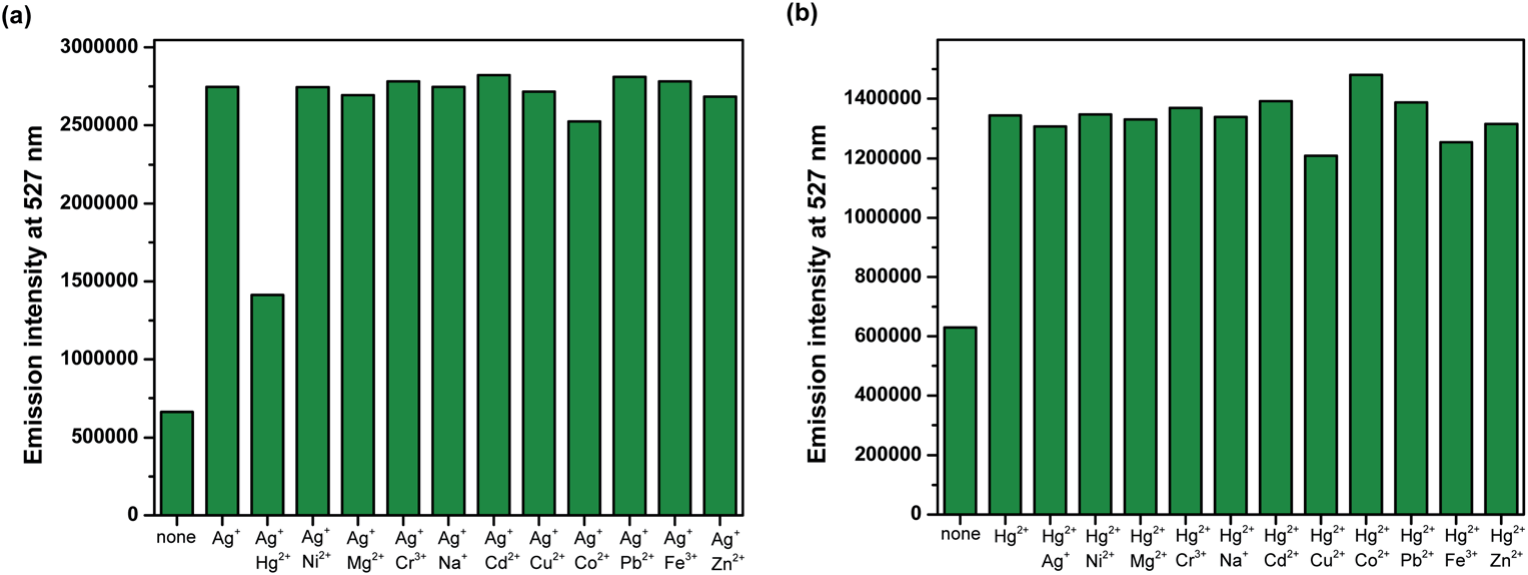
Competitive selectivity of: (a) BODIPY-OO (0.5 μM) toward Ag^+^ (10 equiv.) with other metal ions, and (b) BODIPY-OO (0.5 μM) toward Hg^2+^ (10 equiv.) with other metal ions.

Competition experiments were conducted to investigate high selectivity toward specific analytes over other competitive species by adding 10 equiv. of either Ag^+^ or Hg^2+^ to the solution of BODIPY-OO in the presence of other common metal ions such as Na^+^, Mg^2+^, Fe^3+^, Co^2+^, Ni^2+^, Cu^2+^, Zn^2+^, Cd^2+^, Ag^+^, Pb^2+^, Hg^2+^, and Cr^3+^ (20 equiv.). Fig. 5a clearly revealed no changes in the fluorescence intensity of the BODIPY-OO–Ag^+^ complex upon addition of other metal ions with the exception of Hg^2+^. The addition of Hg^2+^ substantially perturbed the fluorescence emission of BODIPY-OO–Ag^+^ as it reduced about a half of the initial fluorescence intensity. This result implies that the Hg^2+^ ion can interfere the binding between BODIPY-OO and the Ag^+^ ion. A possible factor that affects the higher affinity of the Hg^2+^ ion as compared to Ag^+^ toward the receptor is the smaller ionic diameter (Hg^2+^, 2.0 Å; Ag^+^, 2.3 Å).^70^ Based on the interference experiment for the BODIPY-OO–Hg^2+^ complex, no significant changes occurred for the contact with the other ions (20 equiv.). As shown in Fig. 5b, the specific recognition of Hg^2+^ by BODIPY-OO was observed. When other interfering metal ions were added, there was no interference in the detection of Hg^2+^ from most of the metal ions.

**Fig. 6:**
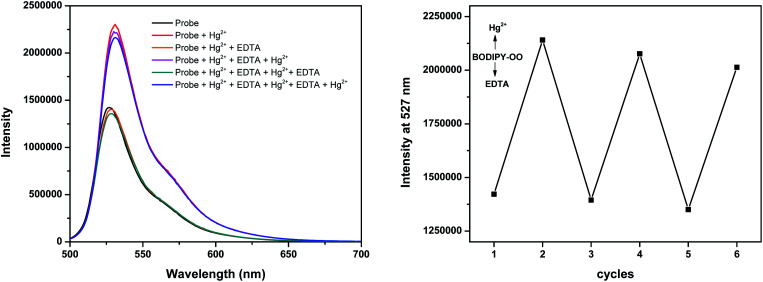
Fluorescence spectral changes of BODIPY-OO (1 μM) after the sequential addition of Hg^2+^ and EDTA.

**Fig. 7:**
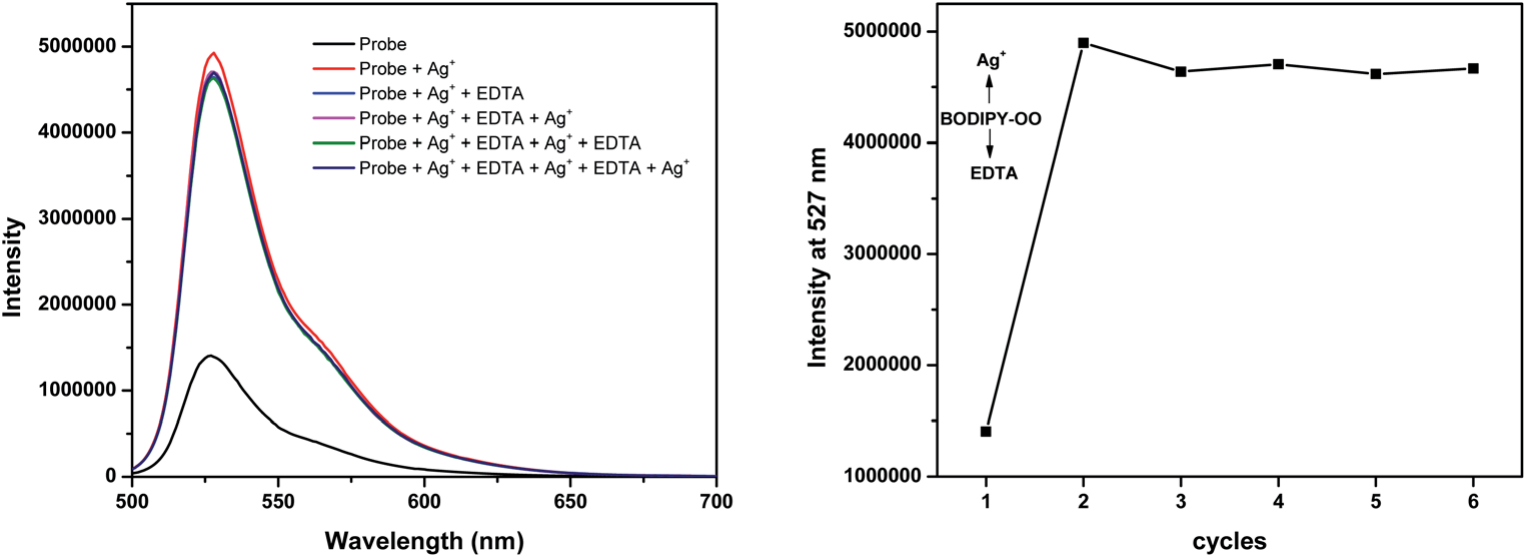
Fluorescence spectral changes of BODIPY-OO (1 μM) after the sequential addition of Ag^+^ and EDTA.

The response behavior of BODIPY-OO toward the Ag^+^ and Hg^2+^ ions in terms of the binding ability and reversibility was further determined to discriminate the Ag^+^ from the Hg^2+^. The discriminative experiment was performed using ethylenediamine tetraacetic acid (EDTA) calcium disodium salt. The addition of EDTA to a solution of BODIPY-OO–Hg^2+^ led to an immediate decrease in the fluorescence intensity (Fig. 6). The intensity can be revived by the addition of Hg^2+^. It was also found that the sequentially alternative addition of Hg^2+^ and EDTA resulted in the reversible emission changes even after several cycles. These results suggested a potential of the probe to be recyclable simply through treatment with a proper reagent such as EDTA. In contrast, the emission changes were not observed for the BODIPY-OO–Ag^+^ complex after the addition of EDTA, (Fig. 7). In other words, the complexation reaction of BODIPY-OO with the Ag^+^ ion was irreversible. This is because EDTA is a good chelating agent for di- and trivalent metal ions and can be seen from the greater formation constant (*K*_f_) for the Hg^2+^–EDTA complex (log(*K*_f_) = 21.5) when compared with the Ag^+^–EDTA complex (log(*K*_f_) = 7.20).^71^ Therefore, the addition of EDTA could assist to distinguish BODIPY-OO–Hg^2+^ from BODIPY-OO–Ag^+^.

### ^1^H-NMR titration for BODIPY-OO with Ag^+^ and Hg^2+^

**Fig. 8:**
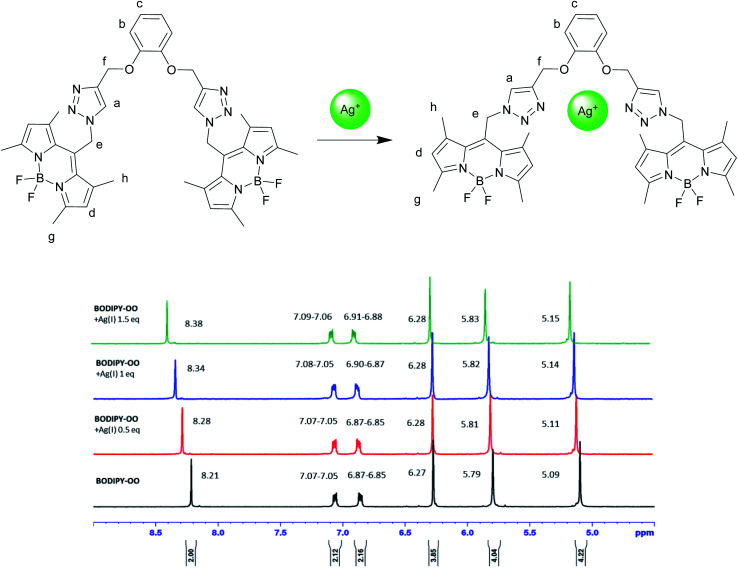
Partial ^1^H NMR titration spectra of BODIPY-OO in DMSO-d_6_ upon addition of Ag^+^ in various amounts.

**Fig. 9:**
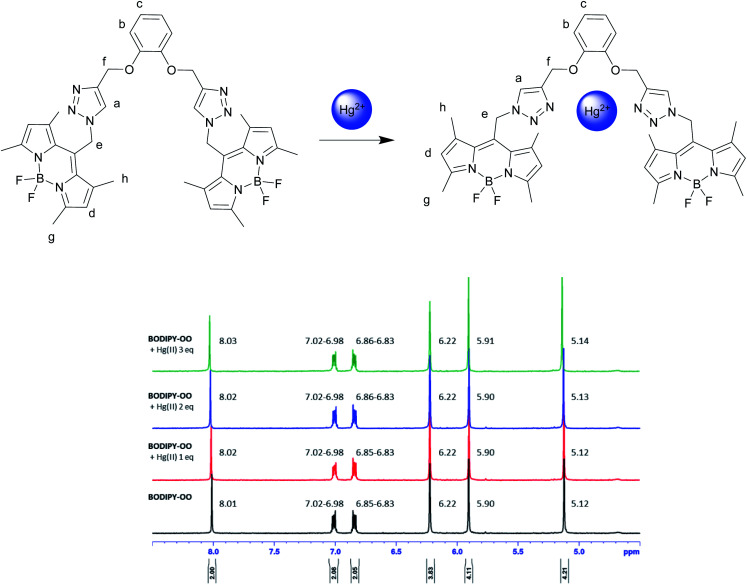
Partial ^1^H NMR titration spectra of BODIPY-OO in acetone-d_6_ upon addition of Hg^2+^ in various amounts.

To understand complexation of BODIPY-OO with the Ag^+^ and Hg^2+^ ions, ^1^H NMR titrations were performed. Fig. 8 shows ^1^H NMR spectra of BODIPY-OO recorded in DMSO-d_6_ with different concentrations of Ag^+^ ranging from 0 to 1.5 equivalents. It was observed that the triazole (H_a_) and methylene protons (H_e_ and H_f_) at *δ* 8.21, 5.79, and 5.09 ppm were obviously downfield shifted to *δ* 8.38, 5.83, and 5.15 ppm, respectively, upon increasing concentrations of the Ag^+^. The downfield shift of the triazole and methylene protons may arise from the interaction of Ag^+^ with the probe through triazole nitrogen and catechol oxygen atoms. In contrast, coordination of BODIPY-OO with the Hg^2+^ changed the chemical shifts of neighboring protons in the binding site very slightly. As shown in Fig. 9, H_a_, H_e_, and H_f_ at *δ* 8.01, 5.90, and 5.12 ppm were shifted to *δ* 8.03, 5.91, and 5.14 ppm, respectively. Very small changes in the ^1^H NMR spectra of BODIPY-OO upon increasing concentrations of the Hg^2+^ are possibly the effect of the rapid quadrupolar relaxation of the Hg^2+^ ion.^72–74^

### Theoretical calculation studies

**Fig. 10:**
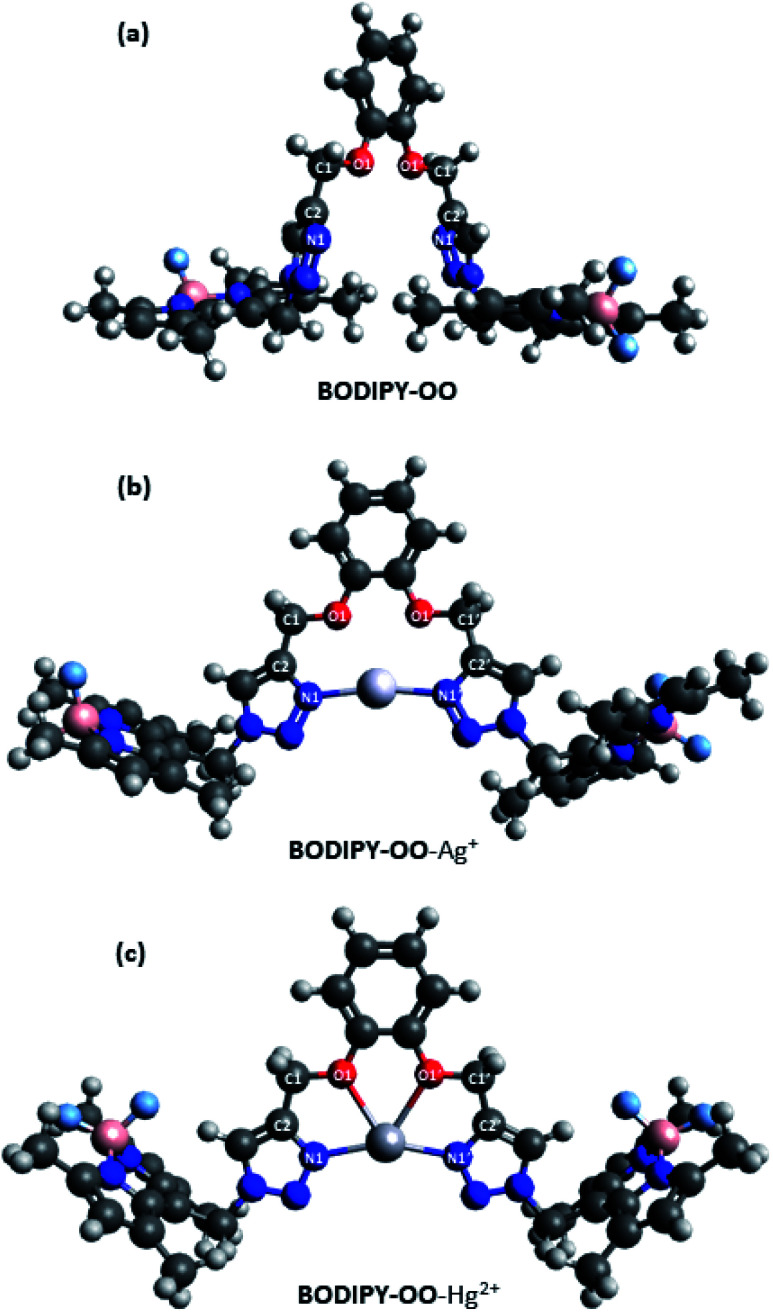
Optimized structures of (a) BODIPY-OO, (b) BODIPY-OO–Ag^+^, and (c) BODIPY-OO–Hg^2+^.

To get insight into the mechanism on the fluorescence sensing of BODIPY-OO toward Ag^+^ and Hg^2+^, density functional theory (DFT) calculations of the sensor and its complexes were performed. Fig. 10 illustrates the calculated energy-minimized structures of BODIPY-OO, BODIPY-OO–Ag^+^, and BODIPY-OO–Hg^2+^ complexes. It can be seen that the triazole units in the free ligand are arranged in a “face-to-face” fashion (Fig. 10a). Dihedral angles of O1–C1–C2–N1 and O1′–C1′–C2′–N1′ are found nearly the same, *i.e.* 114.886° and 114.883°, respectively. On the other hand, BODIPY-OO–Ag^+^ and BODIPY-OO–Hg^2+^ complexes showed significant changes in their geometries. In Fig. 10b, the optimized geometry of BODIPY-OO–Ag^+^ indicates that a stable complex was formed with the lone electron pairs of triazole nitrogen atoms (N1 and N1′) turned toward the bound Ag^+^ ion. The bond lengths of N1–Ag^+^ and N1′–Ag^+^ were 2.19 Å, whereas the dihedral angles of O1–C1–C2–N1 and O1′–C1′–C2′–N1′ were 13.734° and −9.848°, respectively. The bond angle between N1–Ag–N1′ of 168.77° was observed. In contrast, the optimized geometry of BODIPY-OO–Hg^+^ showed that not only N1 and N1′ of the triazole rings but also O1 and O1′ of the catechol unit were involved in the coordination with the Hg^2+^ center with the coordinated bond lengths of 2.22 Å (N1–Hg and N1′–Hg) and 2.48 Å (O1–Hg and O1′–Hg) (Fig. 10c). The calculated bond angle between N1–Hg–N1′ was 157.83°, whereas the dihedral angles of O1–C1–C2–N1 and O1′–C1′–C2′–N1′ were 0.020° and 0.033°, respectively. These results suggested that two coordinated nitrogens are not exactly linear and two triazole rings are almost in the same plane.

Furthermore, calculated frontier molecular orbitals (FMOs) of BODIPY-OO and its complexes are shown in Table S2, ESI,† and their calculated absorption data are summarized in Table 2. The main transition of BODIPY-OO was at 442 nm (*λ*_exp_ = 517 nm) which is related to π → π* transition of the BODIPY units. That of BODIPY-OO–Ag^+^ was found to be very similar at 440 nm (*λ*_exp_ = 517 nm) since there were no significant changes in electronic transition energies of BODIPY-OO and BODIPY-OO–Ag^+^, *i.e.* 2.80 eV and 2.82 eV, respectively. In contrast, the π → π* transition of BODIPY-OO–Hg^2+^ was observed at 456 nm (*λ*_exp_ = 531 nm) which was red-shifted with respect to that of BODIPY-OO and BODIPY-OO–Ag^+^ as a result of the lower energy transition (2.72 eV). These theoretical results are in good agreement with the experimental spectra. On the other hand, comparing with BODIPY-OO–Ag^+^, the lower energies of the HOMOs of BODIPY-OO–Hg^2+^ can be ascribed to a weaker electrostatic repulsion between the metal electrons and lone electron pairs of the triazole and catechol ligands directed toward the metal center.

**Table 2:** Calculated electronic transition energies and oscillator strengths of BODIPY-OO, BODIPY-OO–Ag^+^, and BODIPY-OO–Hg^2+^

Compound	*λ* ^max^_abs_ (nm)	Band assignment	Excitation energy (eV)	Oscillator strength
BODIPY-OO	442	H−0 → L+0 (49%)	2.80	1.21
		H−1 → L+1 (49%)		
BODIPY-OO–Ag^+^	440	H−0 → L+1 (57%)	2.82	1.08
		H−1 → L+0 (41%)		
BODIPY-OO–Hg^2+^	456	H−0 → L+1 (49%)	2.72	1.17
		H−1 → L+0 (49%)		

Notably, the electron density in FMOs of the sensor and its complexes was distributed almost only over the BODIPY units. In other words, the HOMO and the LUMO are heavily biased to the BODIPY units and receive only very minor contributions from the coordination center. As a consequence, the relevant excitation is adequately described as a BODIPY-based π → π* transition with significantly no charge-transfer contributions from the triazole fragments. Therefore, mechanisms like intramolecular charge transfer (ICT) and photoinduced electron transfer (PET) were ruled out.

## Conclusions

We have synthesized a new bifunctional chemosensor BODIPY-OO that can be used as a “turn-on” fluorescent probe for dual detection of Hg^2+^ and Ag^+^ ions. BODIPY-OO can clearly distinguish Hg^2+^ from Ag^+^ by the use of an EDTA. Modifications to chelating sites of this bis-BODIPY linked-triazole platform can not only bring more sensing information but also efficient differentiation of multiple analytes.

## Conflicts of interest

There are no conflicts to declare.

## Supplementary Material

RA-011-D0RA09686E-s001
