# Supplementary material for: Bis-BODIPY linked-triazole based on catechol core for selective dual detection of Ag+ and Hg2+
Source: RSC Adv. 2021 Jan 19;11(6):3703–12. doi: 10.1039/d0ra09686e (PMC8694132; doi:10.1039/d0ra09686e)
Supplement: RA-011-D0RA09686E-s001 [file RA-011-D0RA09686E-s001.pdf]

Electronic Supplementary Material (ESI) for RSC Advances.
This journal is © The Royal Society of Chemistry 2021

Supplementary Information

Bis-BODIPY Linked-Triazole based on Catechol Core for Selective Dual Detection of Ag^+ and Hg^{2+}

Worakrit Saiyasombat,[a] Supavadee Kiatisevi[a,b]*

[a] Department of Chemistry and Center of Excellence for Innovation in Chemistry (PERCH-CIC), Faculty of Science, Mahidol University, Rama VI Rd, Rajthevi, Bangkok 10400, Thailand. E-mail: supavadee.mon@mahidol.edu; Fax: +66-2-354 7151; Tel: +66-2-201 5150

[b] Center of Sustainable Energy and Green Materials, Faculty of Science, Mahidol University, Salaya, Putthamonthon, Nakhon Pathom 73170, Thailand

Contents

1.	Examples of chemosensors for simultaneous detection of Ag^{+} and Hg^{2+}	3
2.	Titration curve and linear relationship between fluorescence intensities of **BODIPY-OO** and concentrations of Ag^{+}	4
3.	Titration curve and linear relationship between fluorescence intensities of **BODIPY-OO** and concentrations of Hg^{2+}	5
4.	Job plots of **BODIPY-OO**-Ag^{+} and **BODIPY-OO**-Hg^{2+} complexes	6
5.	Binding constant (K_a) value for Ag^{+} and Hg^{2+} complexation of **BODIPY-OO**	7
6.	Calculation of emission quantum yields	8
7.	Frontier molecular orbitals (FMOs) and MO energies of **BODIPY-OO**, **BODIPY-OO**-Ag^{+} and **BODIPY-OO**-Hg^{2+}	9
8.	Cartesian coordinates	10–23
9.	NMR spectra	24–27
10.	References	28

1. Examples of chemosensors for simultaneous detection of Ag^{+} and Hg^{2+}

Table S1. Examples of chemosensors for simultaneous detection of Ag^{+} and Hg^{2+}.

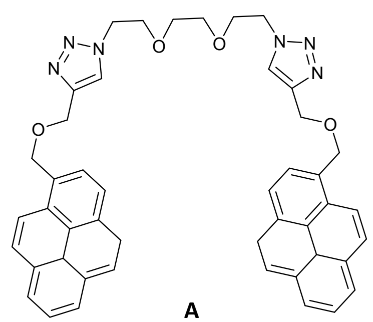

A

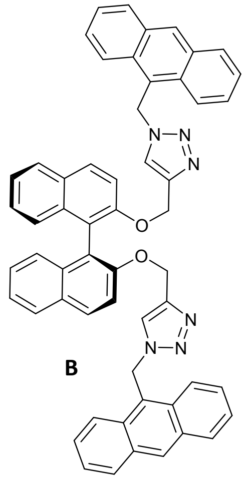

B

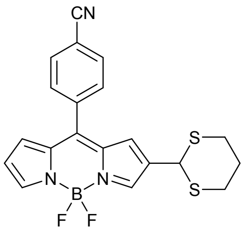

C

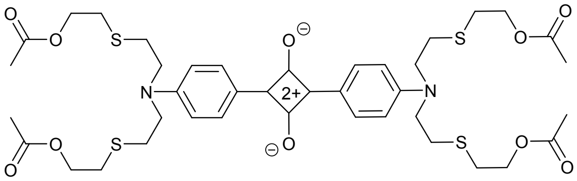

D

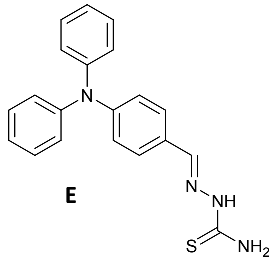

E

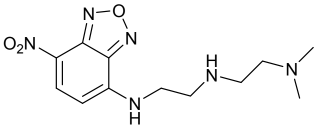

F

Compound	Working system	$\lambda_{ex}/\lambda_{em}$ (nm)	Detection limit Ag^{+}/Hg^{2+}(μM)	Binding constant Ag^{+}/Hg^{2+} (M^{-1})	Operation mode	Ref.
A	CH_3OH	312/478	No data	7.1 x 10^{3}/9.9 x 10^{4}	Turn off	1
B	CH_3OH/H_2O, (200:1, v/v)	367/415	No data	4.1 x 10^{4}/1.0 x 10^{9}	Turn on and Turn off	2
C	THF/water (85:15, v/v)	518/548	140 /650	No data	Turn on	3
D	$EtOH/H_2O$ (50:50, v/v)	652/668	No data/0.13	1.2 x 10^{8}/3.1 x 10^{3}	Turn off	4
E	$DMSO/H_2O$ (9:1, v/v)	375/470	0.59/ 0.19	9.4 x 10^{4}/1.0 x 10^{5}	Turn off	5
F	Buffer/CH_3C N (7:3, v/v)	460/520	0.12/ 0.05	3.5 x 10^{4}/5.0 x 10^{4}	Turn on	6
**BODIPY-OO**	CH_3OH	470/527	0.5/1	1.6× 10^{5}/1.4 × 10^{5}	Turn on	This work

2. Titration curve and linear relationship between fluorescence intensities of **BODIPY-OO** and concentrations of Ag^+

(a)

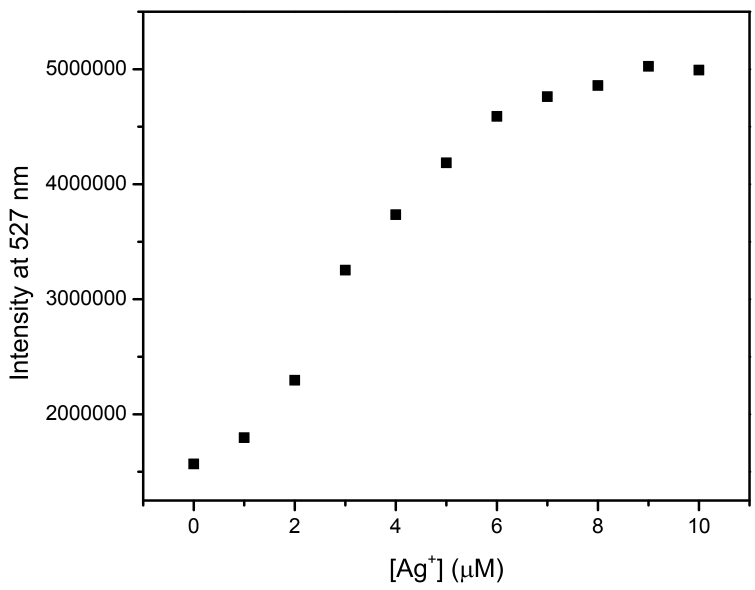

(b)

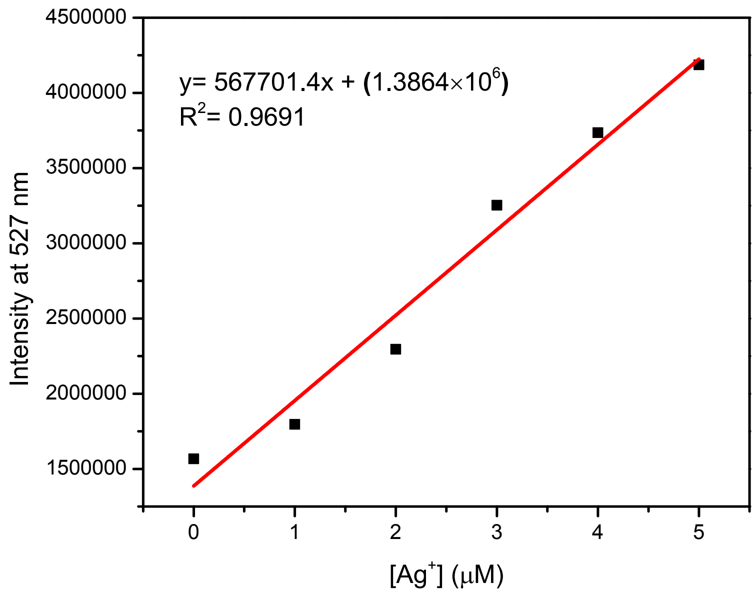

Fig. S1 (a) Titration curve of **BODIPY-OO** with increasing concentrations of Ag^+ ions. (b) Linear relationship between fluorescence intensities of **BODIPY-OO** and concentrations of Ag^+ ions.

3. Titration curve and linear relationship between fluorescence intensities of **BODIPY-OO** and concentrations of Hg^{2+}

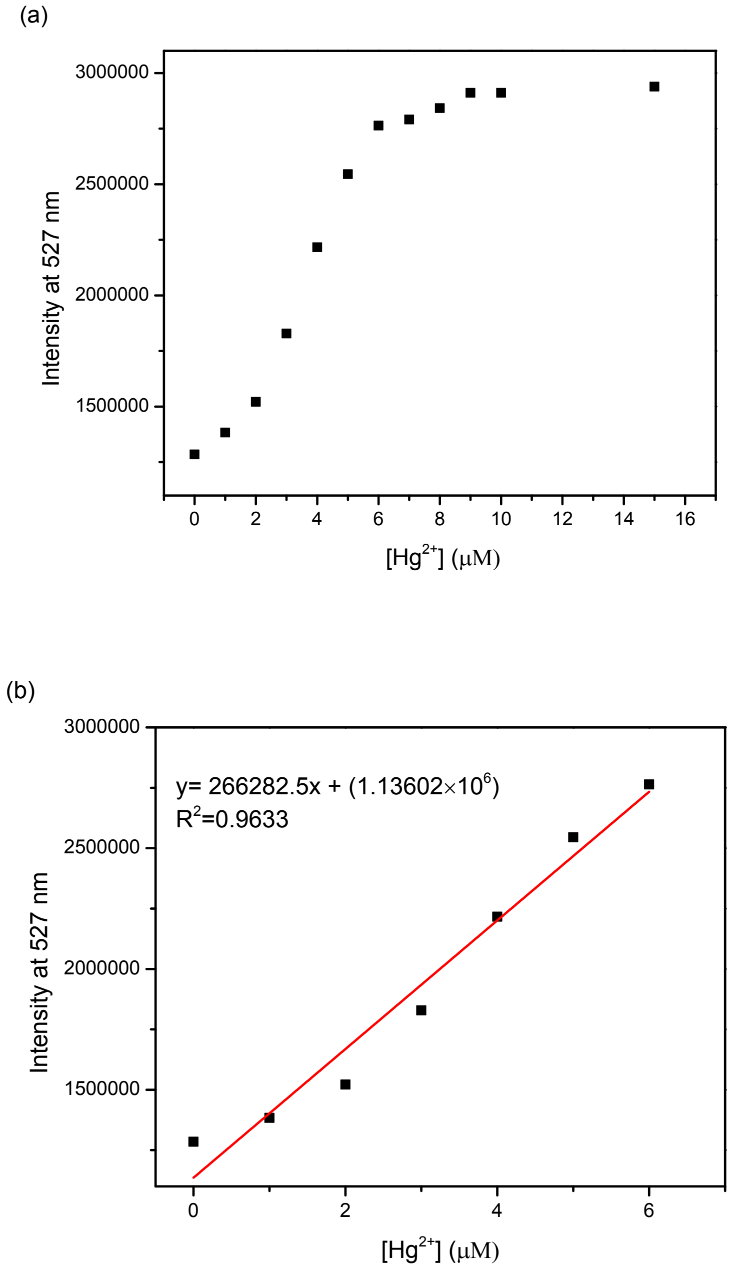

Fig. S2 (a) Titration curve of **BODIPY-OO** with increasing concentrations of Hg^{2+}ions. (b) Linear relationship between fluorescence intensities of **BODIPY-OO** and concentrations of Hg^{2+}ions.

4. Job plotS of **BODIPY-OO**-Ag^{+} and **BODIPY-OO**-Hg^{2+} complexes.

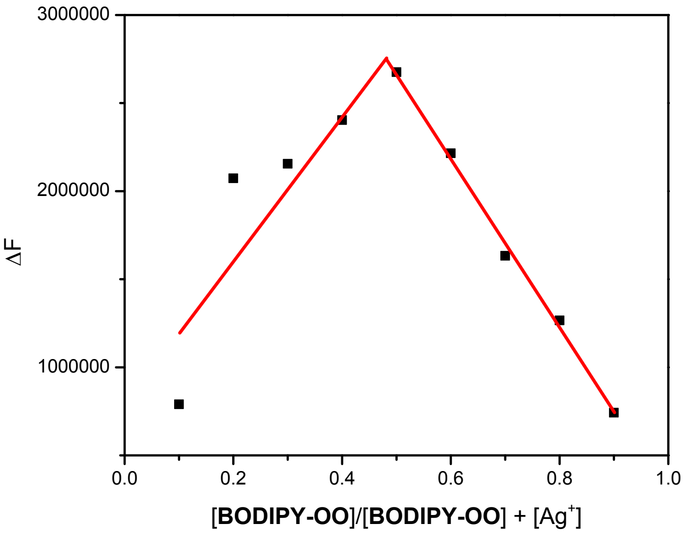

Fig. S3 Job plot of **BODIPY-OO**-Ag^{+} complexes in methanol, indicating the formation of 1:1 complex between Ag^{+} and **BODIPY-OO**.

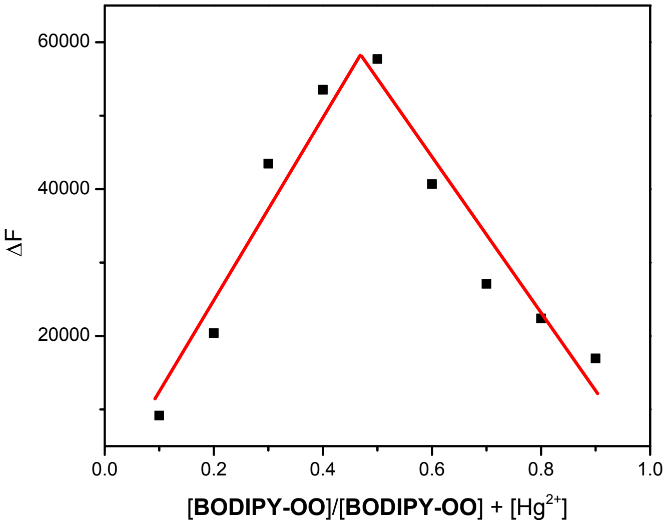

Fig. S4 Job plot of **BODIPY-OO**-Hg^{2+} complexes in methanol, indicating the formation of 1:1 complex between Hg^{2+} and **BODIPY-OO**.

5. Binding constants (K_a) for Ag^+ and Hg^{2+} complexation of **BODIPY-OO**.

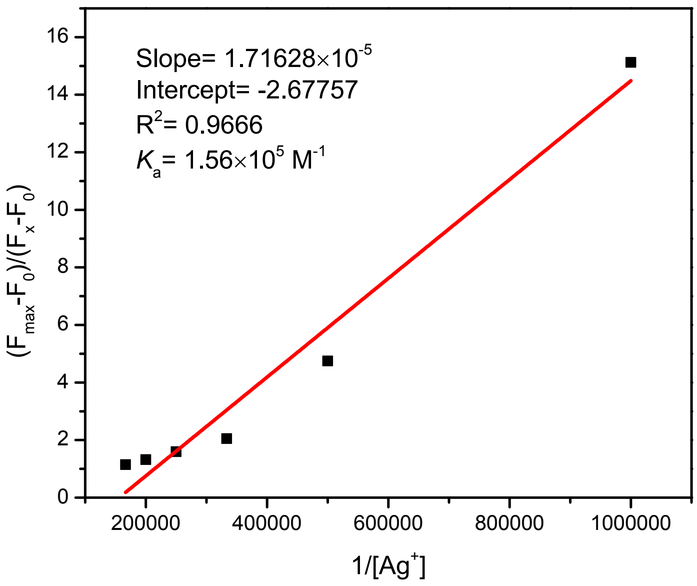

Fig. S5 Binding constant (K_a) value for Ag^+ complexation of **BODIPY-OO**.

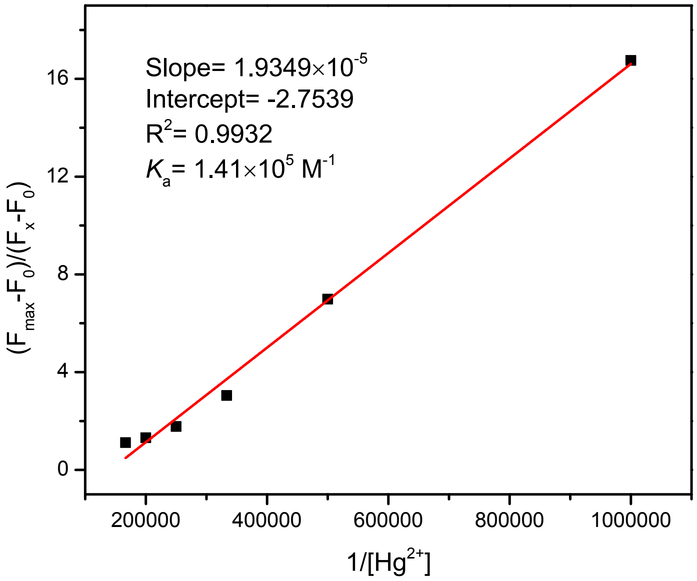

Fig. S6 Binding constant (K_a) value for Hg^{2+} complexation of **BODIPY-OO**.

6. Calculation of emission quantum yields

Emission quantum yield was calculated by using the following equation:

$$\Phi_x = \Phi_{ST}\left(\frac{Grad_X}{Grad_{ST}}\right)\left(\frac{\eta_X^2}{\eta_X^2}\right)$$

Where the subscripts ST and X denote standard and test respectively, Φ is the fluorescence quantum yield, Grad is the gradient obtained from the plot of integrated fluorescence intensity vs absorbance, and η is the refractive index of the solvent.

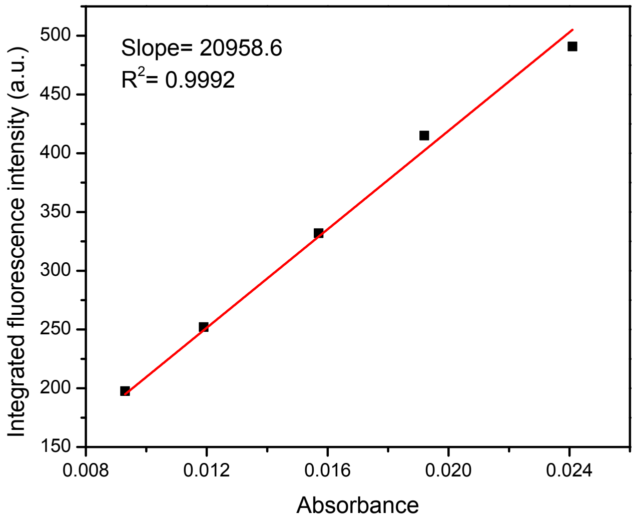

Fig. S7 Plot of integrated fluorescence intensity of fluorescein against absorbance.

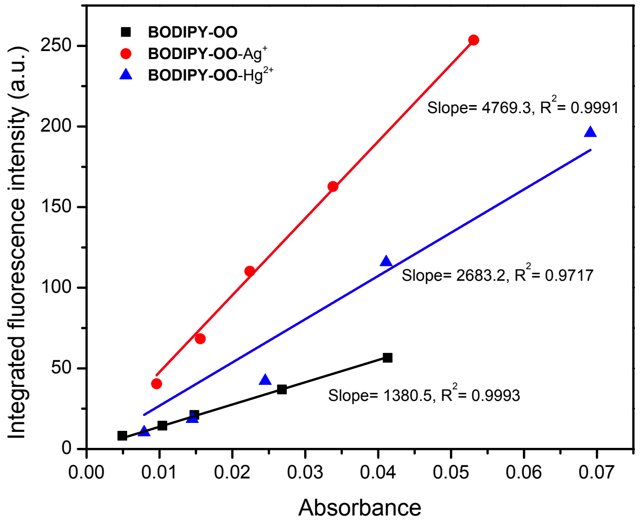

Fig. S8 Plot of integrated fluorescence intensity of **BODIPY-OO** and its complexes against absorbance.

7. Frontier molecular orbitals (MOs) and MO energies of **BODIPY-OO**, **BODIPY-OO**-Ag^{+} and **BODIPY-OO**-Hg^{2+}

Table S2. Frontier molecular orbitals (MOs) and MO energies of **BODIPY-OO**, **BODIPY-OO**-Ag^{+} and **BODIPY-OO**-Hg^{2+}

**BODIPY-OO**	**BODIPY-OO**-Ag^{+}	**BODIPY-OO**-Hg^{2+}
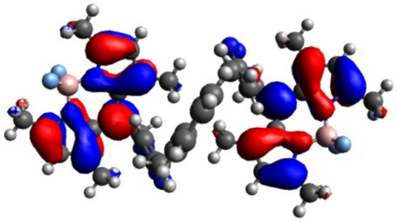	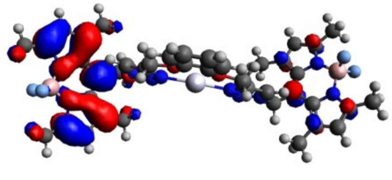	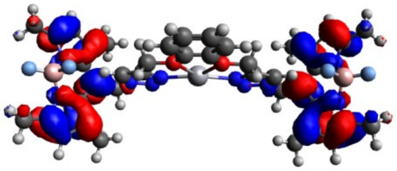
L+1, E = -1.800 eV	L+1, E = -2.094	L+1, E = -2.261
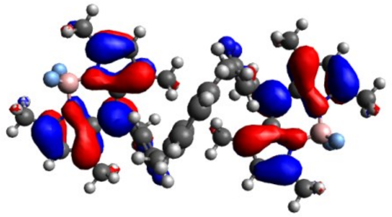	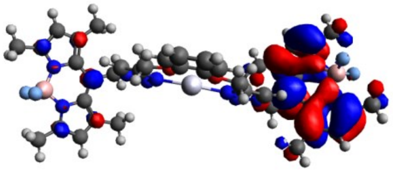	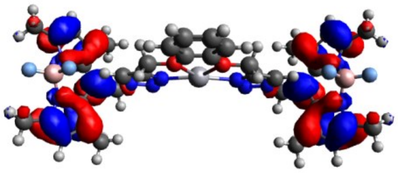
L+0, E = -1.807 eV	L+0, E = -2.097 eV	L+0, E = -2.269 eV
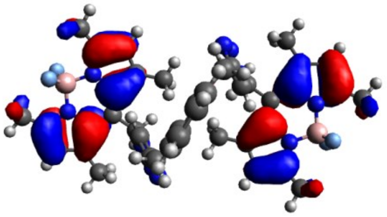	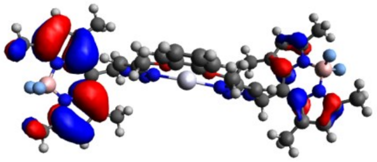	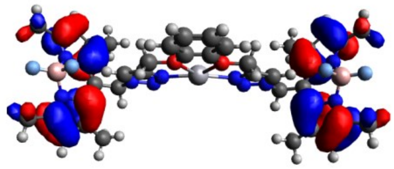
H-0, E = -6.855 eV	H-0, E = -7.117 eV	H-0, E = -7.194 eV
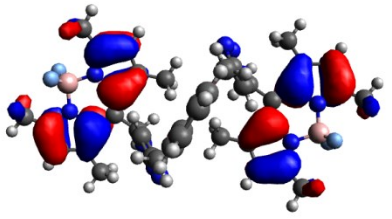	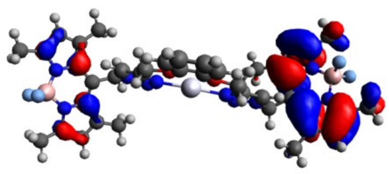	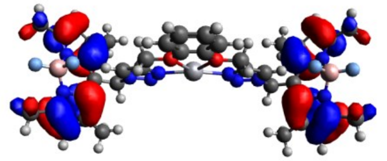
H-1, E = -6.858 eV	H-1, E = -7.118 eV	H-1, E = -7.194 eV

8. Cartesian coordinates

Cartesian coordinates of optimized structures

BODIPY-OO

F	7.41805900	-2.22939000	1.85043500
F	7.35114000	0.02991300	1.48179900
N	6.65960100	-1.44297000	-0.30678200
C	4.23730000	-1.51257700	-0.44625100
N	5.25570600	-1.15244600	1.72947600
C	4.10990800	-1.29642200	0.93830000
C	5.49398000	-1.59154400	-1.06745000
C	5.89158100	-1.81023100	-2.43486200
C	7.27918800	-1.77879600	-2.43680000
C	2.96585800	-1.17423700	1.80788100
C	4.88356100	-0.95277300	3.00795600
C	7.72756000	-1.55293000	-1.11861100
C	3.47538100	-0.96077300	3.08132600
C	5.86569900	-0.75905000	4.11582300
C	9.13256500	-1.44139000	-0.62568900
B	6.73117700	-1.19078300	1.22857200
C	1.49387900	-1.24932600	1.51030800
C	5.06154300	-2.05293000	-3.66299000

N	1.60196500	0.89294300	-3.32221800
N	2.56180200	-0.35458100	-1.86295800
N	1.97830400	-0.33628000	-3.08879000
C	2.55419200	0.88151600	-1.31167200
C	1.93813000	1.67531500	-2.25835200
C	2.98473000	-1.63701500	-1.28852200
C	1.58989600	3.12542400	-2.16948800
C	0.37764200	4.49862100	-0.60047200
C	0.73791800	5.70667100	-1.18939400
C	-0.37793900	4.49872300	0.59987400
C	0.36490300	6.91966700	-0.59075500
C	-0.73824000	5.70686900	1.18858200
C	-0.36524300	6.91976600	0.58973200
C	-1.59020300	3.12582800	2.16911700
H	7.92016500	-1.90602000	-3.29980900
H	2.89576400	-0.81934800	3.98442500
H	6.50976200	0.10253300	3.91078200
H	6.52367900	-1.63031200	4.20174500
H	5.34659200	-0.60397200	5.06473700
H	9.29468600	-0.47010400	-0.14643600
H	9.83890500	-1.55885300	-1.45113900
H	9.33378700	-2.20387500	0.13420100
H	0.92085400	-1.00505000	2.40948900

H	1.18699800	-2.25622500	1.19624300
H	1.18089200	-0.54614200	0.73004400
H	5.70979400	-2.06565200	-4.54430900
H	4.29303900	-1.29076800	-3.82616900
H	4.55213200	-3.02574300	-3.62879300
H	2.96559000	1.09286700	-0.33736400
H	2.16439800	-2.04035200	-0.69625300
H	3.13275900	-2.30553400	-2.13347800
H	1.13030200	3.45080400	-3.11132000
H	2.48164800	3.74066900	-1.98570200
H	1.29897100	5.71465400	-2.11774300
H	0.65208600	7.85578700	-1.06060800
H	-1.29931300	5.71499700	2.11691900
H	-0.65243800	7.85596600	1.05941800
H	-1.13069500	3.45148600	3.11089300
H	-2.48198800	3.74097300	1.98512200
F	-7.35095100	0.02952300	-1.48215000
F	-7.41757500	-2.22982300	-1.85057800
N	-5.25535800	-1.15269400	-1.72933200
C	-4.23730300	-1.51252800	0.44660900
N	-6.65957900	-1.44309400	0.30671200
C	-5.49408200	-1.59139900	1.06761300
C	-4.10968500	-1.29658000	-0.93795400

C	-2.96549400	-1.17454000	-1.80738000
C	-3.47481800	-0.96121900	-3.08092500
C	-5.89189000	-1.80991200	2.43498100
C	-7.72767500	-1.55305700	1.11837200
C	-4.88301200	-0.95320600	-3.00777600
C	-7.27950600	-1.77864800	2.43667400
C	-9.13260400	-1.44163900	0.62520200
C	-5.86497000	-0.75956700	-4.11581300
B	-6.73091500	-1.19107300	-1.22867700
C	-5.06202800	-2.05227600	3.66329700
C	-1.49355400	-1.24957500	-1.50960800
N	-1.60229200	0.89359400	3.32239900
N	-2.56198800	-0.35427100	1.86333700
N	-1.97859100	-0.33568500	3.08921600
C	-2.55433500	0.88170100	1.31176900
C	-1.93837800	1.67572400	2.25832600
C	-2.98484000	-1.63679900	1.28906900
H	-2.89507000	-0.81993000	-3.98396100
H	-7.92060900	-1.90579700	3.29960100
H	-9.33334900	-2.20353300	-0.13539400
H	-9.29497300	-0.46999500	0.14673900
H	-9.83909700	-1.56003000	1.45038600
H	-6.52308900	-1.63073100	-4.20163400

H	-5.34571300	-0.60477500	-5.06469400
H	-6.50891400	0.10217300	-3.91104900
H	-5.71037500	-2.06454500	4.54455100
H	-4.29340400	-1.29020000	3.82626800
H	-4.55279400	-3.02520200	3.62951300
H	-0.92041900	-1.00540500	-2.40875000
H	-1.18667900	-2.25642400	-1.19537900
H	-1.18066200	-0.54628300	-0.72940300
H	-2.96568000	1.09284200	0.33739400
H	-2.16444000	-2.04020600	0.69694500
H	-3.13294600	-2.30520100	2.13411100
O	-0.67989400	3.25614200	1.07653500
O	0.67961600	3.25595700	-1.07689900

BODIPY-OO-Ag^+

F	10.32118800	-1.28663500	-0.60098400
F	9.26015300	0.77808000	-0.87035600
N	8.02248500	-1.25498500	-1.41911000
C	6.35650900	-1.59421500	0.32451300
N	8.53080900	-0.68159800	0.94440100
C	7.23778200	-1.11215100	1.31149900
C	6.73869400	-1.68986000	-1.02167100
C	6.07596800	-2.19423500	-2.20591100
C	6.97892700	-2.03819700	-3.25448600

C	7.13374700	-0.96220100	2.74658800
C	9.20647200	-0.28314000	2.05445200
C	8.17113500	-1.45607900	-2.75410000
C	8.35833700	-0.45133400	3.17616700
C	10.60090000	0.23963400	2.01063700
C	9.41061600	-1.09114200	-3.49487400
B	9.07207100	-0.59726600	-0.49916300
C	6.00092300	-1.29203600	3.67993400
C	4.71548700	-2.81293800	-2.36007800
N	2.13456300	0.14299200	0.15389000
N	4.01611300	-0.82547000	0.56280200
N	2.72689900	-1.05859800	0.18338900
C	4.23834600	0.50528100	0.77898900
C	3.02802900	1.12497000	0.51380200
C	4.94587200	-1.97132800	0.72304800
C	2.68496200	2.58173900	0.54496800
C	0.65704600	3.95951000	0.42411500
C	1.35398100	5.15298900	0.60190900
C	-0.73294800	3.98335800	0.18663500
C	0.66903200	6.37696100	0.54822000
C	-1.41003700	5.20031500	0.13565300
C	-0.70510500	6.40037900	0.31779500
C	-2.73397400	2.67807000	-0.37150000

H	6.81220700	-2.31901000	-4.28351200
H	8.62917900	-0.22631600	4.19684500
H	10.67073200	1.09109900	1.32445800
H	11.28769500	-0.52280700	1.62784700
H	10.92728600	0.55097200	3.00485400
H	9.63764700	-0.02726200	-3.36199700
H	9.30143000	-1.30522800	-4.55973400
H	10.27112700	-1.64214100	-3.10077300
H	6.26419500	-0.98653900	4.69643700
H	5.79160800	-2.36919200	3.71893400
H	5.06481200	-0.77939600	3.42478100
H	4.52538900	-3.02183600	-3.41651000
H	3.90374600	-2.16915700	-2.00332100
H	4.63509000	-3.77141900	-1.82921100
H	5.19751500	0.89336200	1.07473400
H	4.89831200	-2.29983600	1.75908600
H	4.54174900	-2.76655700	0.10264700
H	3.14070500	3.11051500	-0.30345800
H	3.05179200	3.03987100	1.47195200
H	2.42167700	5.14805700	0.78299800
H	1.21759500	7.30113900	0.68780600
H	-2.47792000	5.23194000	-0.04150900
H	-1.23842700	7.34295500	0.27716300

H	-3.36312400	3.11611200	0.41360800
H	-2.88448000	3.25458700	-1.29458800
F	-9.10653200	0.46891500	1.46710700
F	-10.20886700	-1.49830500	0.85472800
N	-8.61069100	-0.51932100	-0.71001000
C	-6.38942000	-1.51719500	-0.58313000
N	-7.82740700	-1.60944500	1.38034700
C	-6.60505100	-1.91851200	0.74349000
C	-7.37946600	-0.84016500	-1.32107300
C	-7.45058700	-0.37044300	-2.68743400
C	-8.70992800	0.20992800	-2.83658200
C	-5.80586200	-2.65972600	1.69607800
C	-7.81366700	-2.10750800	2.64376200
C	-9.41094700	0.11092800	-1.61001100
C	-6.57320300	-2.76165200	2.85366400
C	-8.95192100	-1.94350400	3.59005600
C	-10.78129800	0.59142500	-1.27744300
B	-8.97485200	-0.77956100	0.76758700
C	-4.43822900	-3.26267000	1.54398200
C	-6.44773900	-0.47112300	-3.80421700
N	-2.15744300	0.22373600	-0.60930800
N	-4.08971000	-0.67213700	-0.93926600
N	-2.76010500	-0.95364000	-0.82552000

C	-4.32997600	0.66484500	-0.79173700
C	-3.08635200	1.23761300	-0.57982500
C	-5.04360100	-1.77287700	-1.22657800
H	-9.10053200	0.65540600	-3.73919900
H	-6.28357700	-3.26079800	3.76609100
H	-9.85509200	-2.41813500	3.19186500
H	-9.19237200	-0.88228600	3.71953100
H	-8.71300500	-2.38011300	4.56161700
H	-11.42307600	-0.24701000	-0.98664500
H	-11.22697100	1.10777400	-2.12985100
H	-10.75213600	1.27084700	-0.41820600
H	-4.11925100	-3.69330600	2.49720300
H	-3.67635100	-2.53472600	1.24321800
H	-4.42481500	-4.07865000	0.80854100
H	-6.83065900	0.04814500	-4.68718400
H	-6.26176500	-1.51037100	-4.10580800
H	-5.47924100	-0.01620800	-3.56111500
H	-5.31980800	1.08497400	-0.83241600
H	-5.12986400	-1.86702100	-2.30703000
H	-4.57274500	-2.67705200	-0.85066100
O	-1.33785900	2.73260400	0.01334000
O	1.24120100	2.68783600	0.46421700
Ag	-0.00961400	0.39752600	-0.21752500

BODIPY-OO-Hg^{2+}

F	-9.59685600	0.95129000	0.00069400
F	-7.25843100	1.32551200	0.00068300
N	-8.10997000	-0.57111200	1.23360100
C	-6.44033000	-1.82978800	-0.00052800
N	-8.11010600	-0.57019000	-1.23354600
C	-7.02813000	-1.47858100	-1.22994000
C	-7.02799300	-1.47949700	1.22921000
C	-6.83740500	-1.92690100	2.58918600
C	-7.81859500	-1.28883200	3.34663500
C	-6.83768400	-1.92496900	-2.59027600
C	-8.58093300	-0.43601000	-2.50233900
C	-8.58067900	-0.43789500	2.50253700
C	-7.81892900	-1.28630700	-3.34715200
C	-9.69273600	0.47900600	-2.88273600
C	-9.69255100	0.47671600	2.88371000
B	-8.33893600	0.32310700	0.00037300
C	-5.87378100	-2.94174300	-3.13305400
C	-5.87338900	-2.94402700	3.13109800
N	-2.18177500	-0.25248100	0.00026400
N	-4.08322000	-1.22866000	-0.00027500
N	-2.75242800	-1.47230100	-0.00023200
C	-4.36676100	0.11412000	0.00018200

C	-3.13064200	0.73886500	0.00052100
C	-5.04232300	-2.39226800	-0.00080600
C	-2.78042600	2.19453900	0.00122500
C	-0.70269600	3.55612500	0.00052300
C	-1.40582100	4.75737500	0.00106800
C	0.70272800	3.55612700	-0.00024800
C	-0.69752600	5.96821400	0.00076700
C	1.40584700	4.75738200	-0.00057800
C	0.69754600	5.96821700	-0.00007000
C	2.78045700	2.19453000	-0.00099000
H	-7.99311400	-1.42460600	4.40335700
H	-7.99357200	-1.42131100	-4.40395100
H	-9.44149700	1.52439000	-2.66869100
H	-10.59854600	0.25909600	-2.30913400
H	-9.91423200	0.38440900	-3.94735100
H	-9.44206100	1.52215800	2.66916400
H	-9.91310800	0.38221600	3.94852800
H	-10.59877400	0.25618900	2.31096500
H	-6.08227100	-3.12461300	-4.19034100
H	-5.95733700	-3.90830600	-2.62054700
H	-4.82741100	-2.61404600	-3.06737400
H	-6.08156300	-3.12748200	4.18834600
H	-4.82702100	-2.61636000	3.06526800

H	-5.95716500	-3.91029700	2.61806800
H	-5.36923600	0.53037000	0.00024800
H	-4.81408900	-2.98036200	-0.88675400
H	-4.81404000	-2.98120600	0.88457200
H	-3.17297700	2.69500000	0.89328500
H	-3.17371400	2.69601700	-0.88993000
H	-2.48831500	4.77375400	0.00173700
H	-1.24432000	6.90327300	0.00118800
H	2.48834000	4.77376800	-0.00124000
H	1.24433500	6.90328000	-0.00033100
H	3.17312900	2.69517100	-0.89289300
H	3.17363600	2.69582000	0.89032200
F	7.25849400	1.32554900	-0.00029900
F	9.59690300	0.95122000	-0.00006400
N	8.10992700	-0.57043400	1.23367700
C	6.44031600	-1.82979200	0.00019100
N	8.11011200	-0.57087300	-1.23347800
C	7.02812600	-1.47925100	-1.22940300
C	7.02795500	-1.47882800	1.22974700
C	6.83733400	-1.92549200	2.58996600
C	7.81847400	-1.28697700	3.34710000
C	6.83769900	-1.92637500	-2.58949200
C	8.58097600	-0.43740000	-2.50233000

C	8.58058800	-0.43651100	2.50255800
C	7.81896500	-1.28813600	-3.34669800
C	9.69290200	0.47727900	-2.88318200
C	9.69235700	0.47841200	2.88327800
B	8.33895500	0.32308900	-0.00003700
C	5.87374800	-2.94338400	-3.13173800
C	5.87335900	-2.94237700	3.13240700
N	2.18178400	-0.25248300	-0.00033300
N	4.08321500	-1.22868200	-0.00009100
N	2.75242000	-1.47231200	-0.00000400
C	4.36677300	0.11409500	-0.00036100
C	3.13066200	0.73885200	-0.00054100
C	5.04231600	-2.39228900	0.00018800
H	7.99297800	-1.42218900	4.40389500
H	7.99360100	-1.42368500	-4.40342900
H	10.59904500	0.25665200	-2.31034900
H	9.44237900	1.52268400	-2.66848400
H	9.91360500	0.38297200	-3.94798600
H	10.59830500	0.25842800	2.30991600
H	9.91356900	0.38377000	3.94794900
H	9.44127300	1.52381800	2.66917800
H	6.08206300	-3.12662400	-4.18899600
H	4.82737400	-2.61572000	-3.06598000

H	5.95744400	-3.90975900	-2.61889600
H	6.08167300	-3.12542100	4.18969900
H	5.95702400	-3.90885200	2.61974900
H	4.82699200	-2.61469700	3.06660100
H	5.36925600	0.53032800	-0.00042900
H	4.81400500	-2.98063000	0.88595300
H	4.81411300	-2.98098100	-0.88537500
O	1.31791000	2.27958900	-0.00064500
O	-1.31787700	2.27958400	0.00071700
Hg	0.00000400	0.17483000	-0.00007300

9. NMR spectra

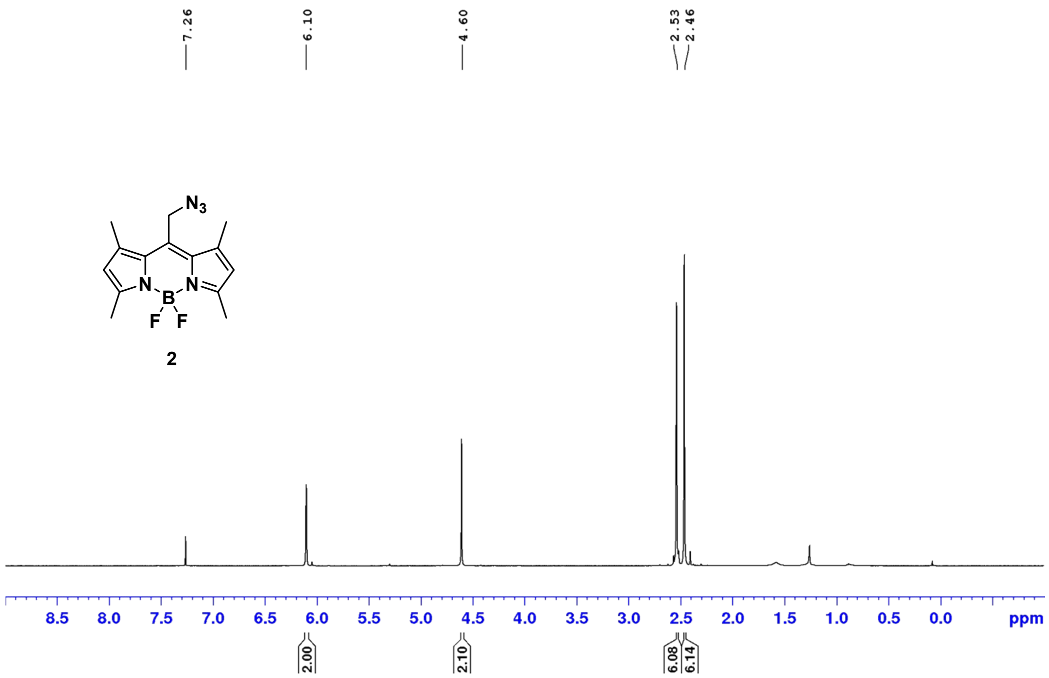

Fig. S9 ^{1}H NMR (400 MHz) of **2** in $CDCl_3$.

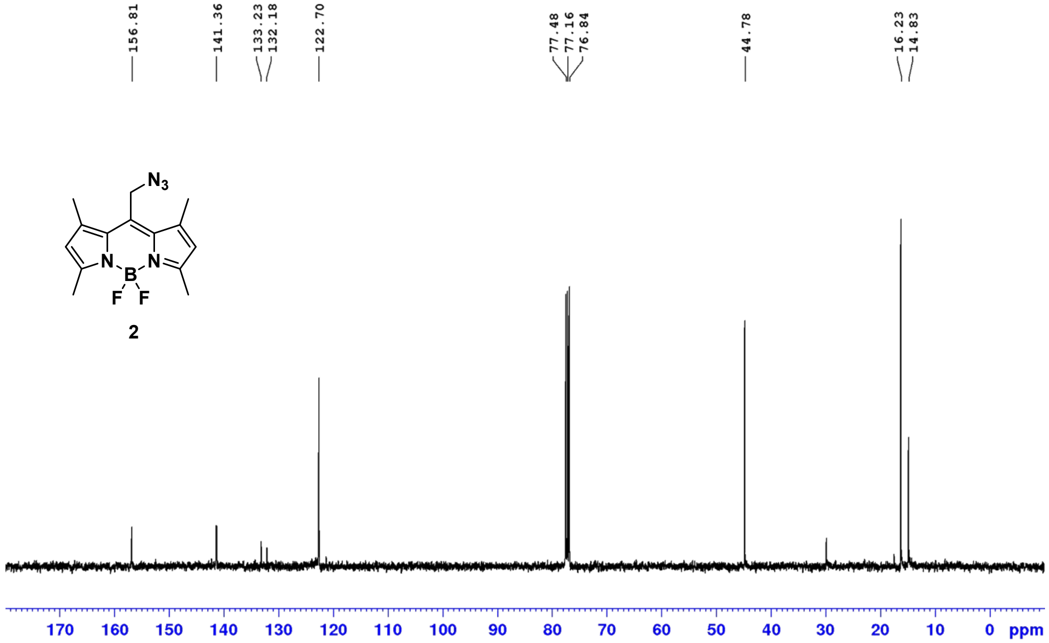

Fig. S10 ^{13}C NMR (100 MHz) of **2** in $CDCl_3$.

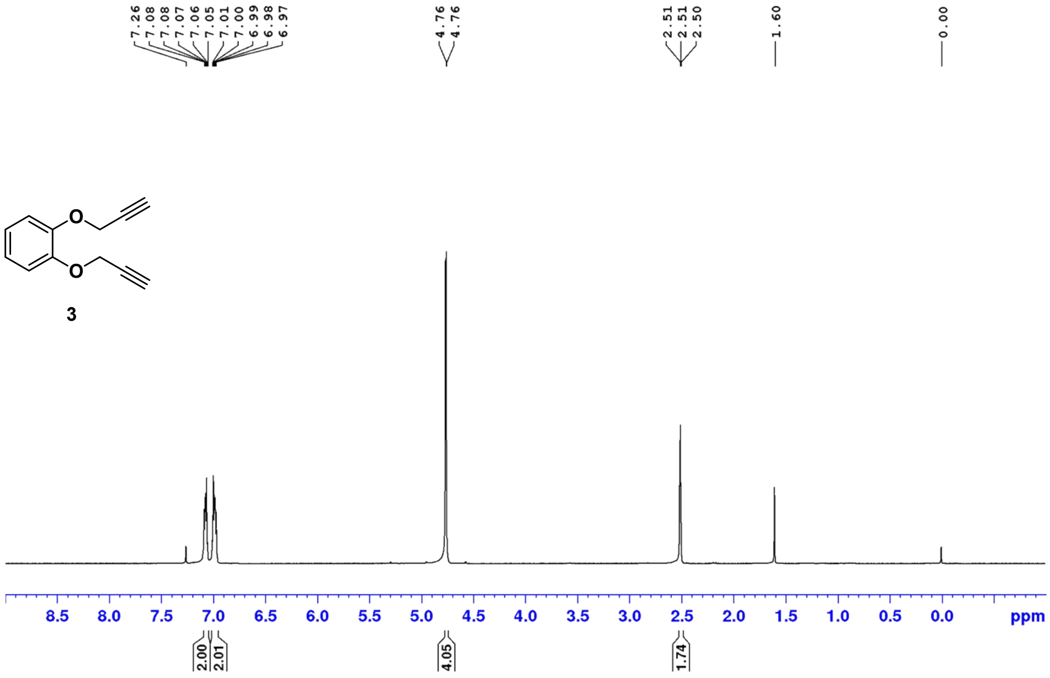

Fig. S11 ^{1}H NMR (400 MHz) of **3** in $CDCl_3$.

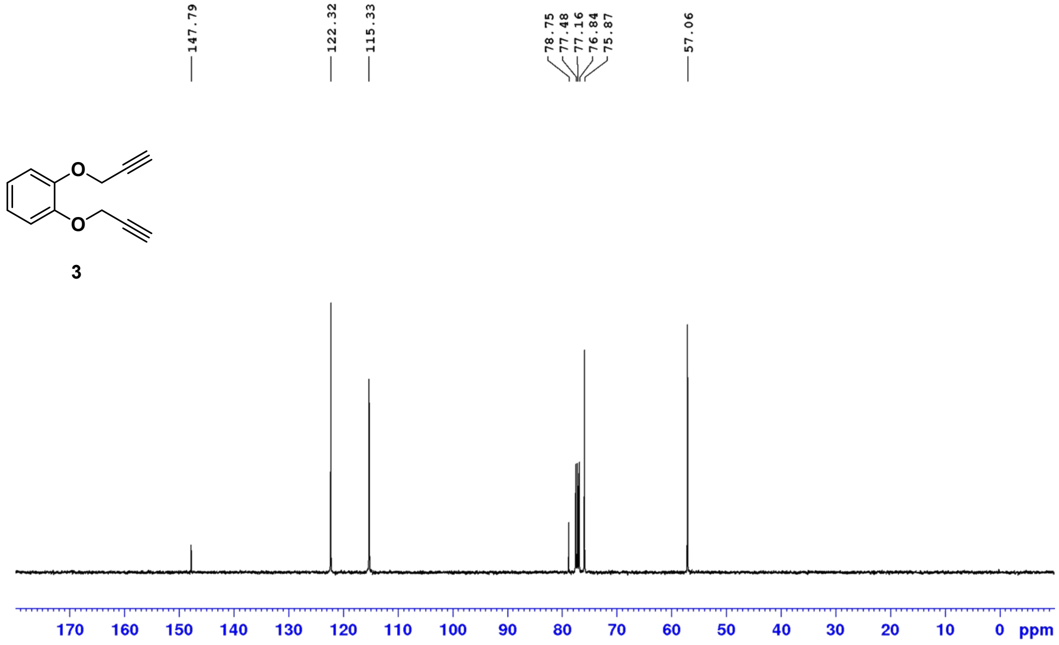

Fig. S12 ^{13}C NMR (100 MHz) of **3** in $CDCl_3$.

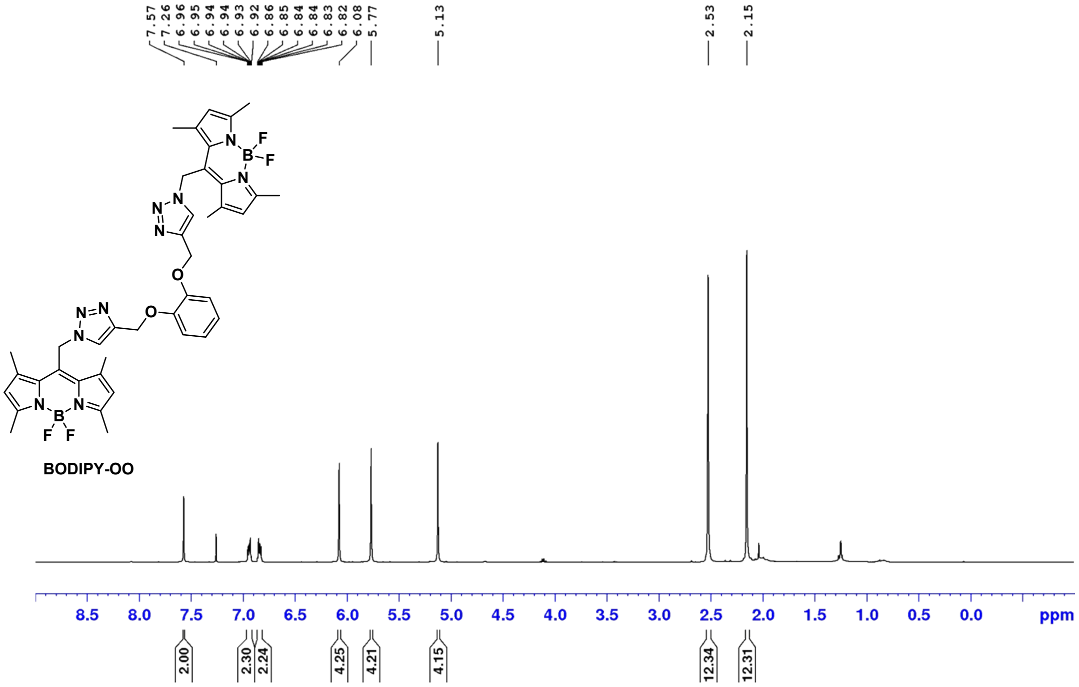

Fig. S13 ^{1}H NMR (400 MHz) of **BODIPY-OO** in $CDCl_3$.

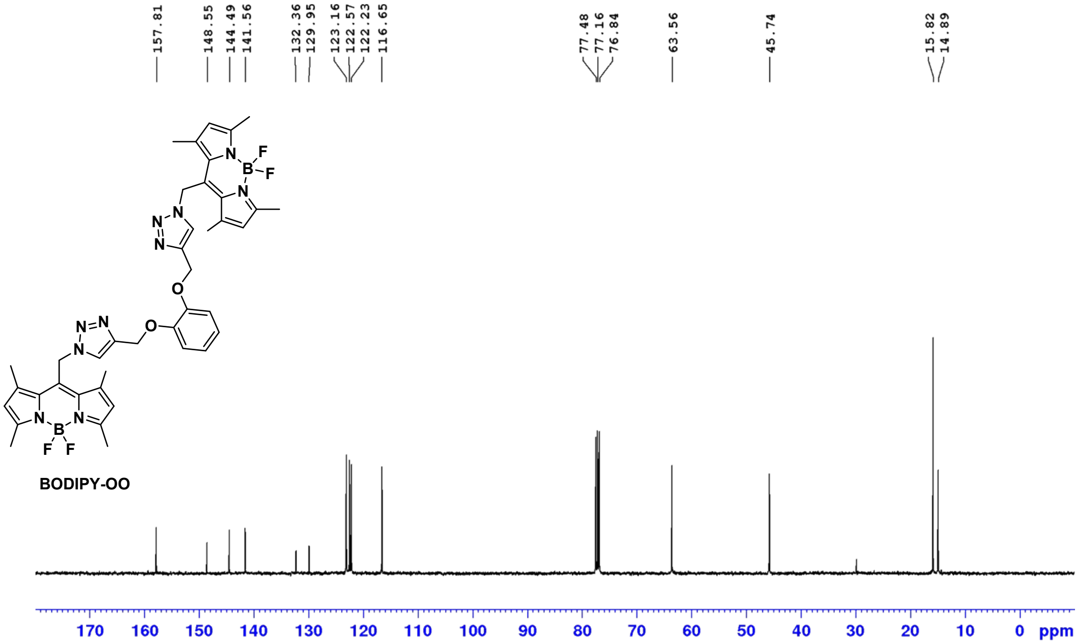

Fig. S14 ^{13}C NMR (100 MHz) of **BODIPY-OO** in $CDCl_3$.

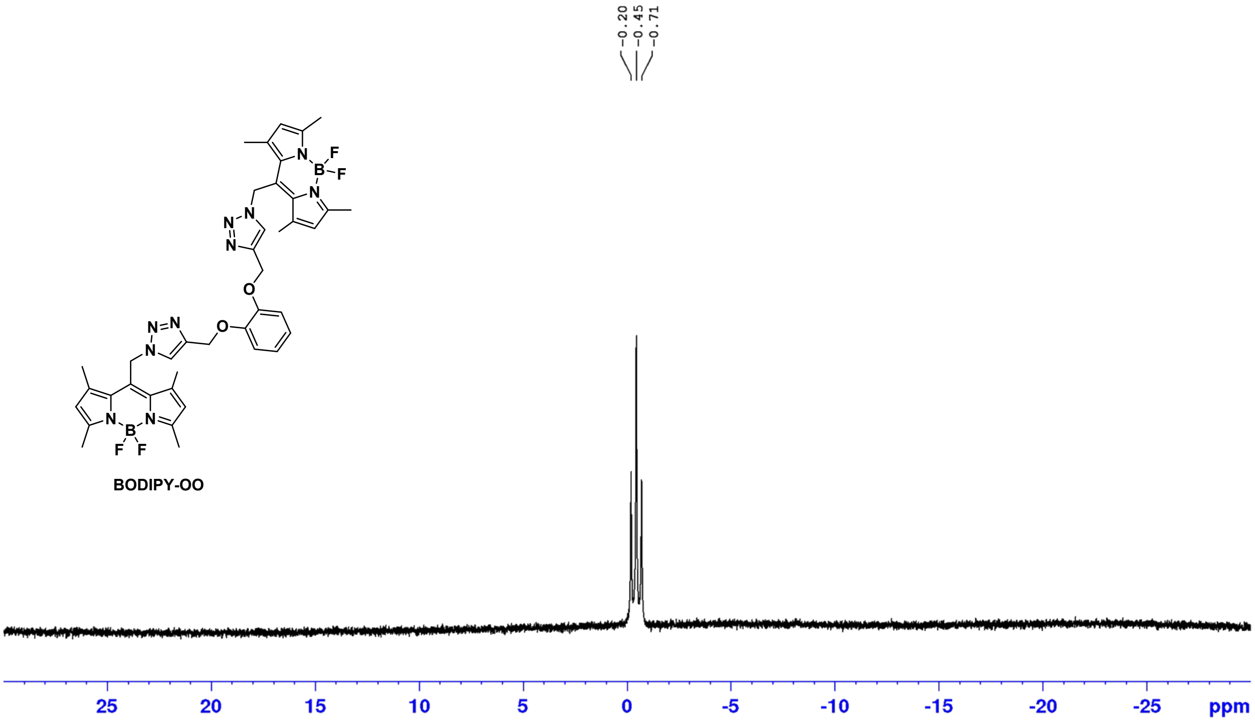

Fig. S15 ^{11}B NMR (128 MHz) of **BODIPY-OO** in $CDCl_3$.

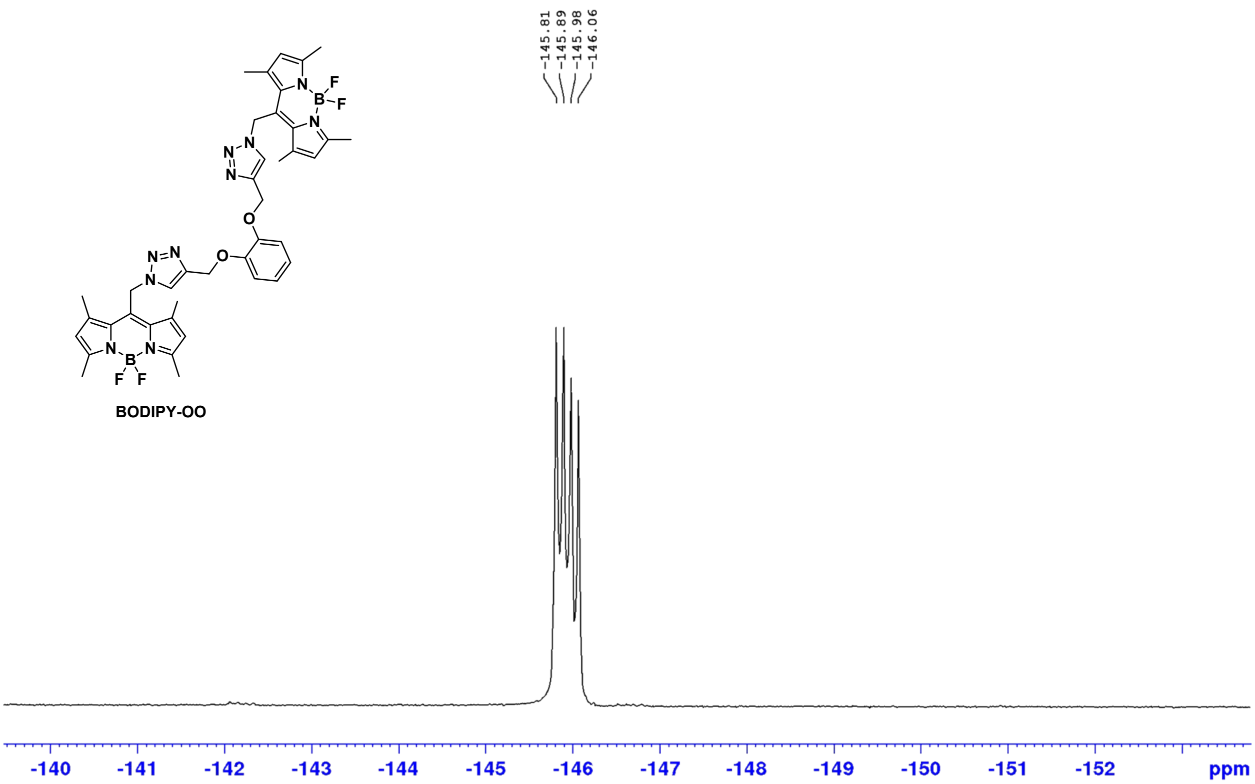

Fig. S16 ^{19}F NMR (377 MHz) of **BODIPY-OO** in $CDCl_3$.

10. References

1. H.-C. Hung, C.-W. Cheng, Y.-Y. Wang, Y.-J. Chen and W.-S. Chung, *Eur. J. Org. Chem.*, 2009, **2009**, 6360–6366.
2. X. Liu, X. Yang, H. Peng, C. Zhu and Y. Cheng, *Tetrahedron Lett.*, 2011, **52**, 2295–2298.
3. X. Zhang, Y. Xu, P. Guo and X. Qian, *New J. Chem.*, 2012, **36**, 1621–1625.
4. J. Fan, C. Chen, Q. Lin and N. Fu, *Sens. Actuators B Chem.*, 2012, **173**, 874–881.
5. W. Shi, Y. Chen, X. Chen, Z. Xie and Y. Hui, *J. Lumin.*, 2016, **174**, 56–62.
6. S. Y. Lee, K. H. Bok and C. Kim, *RSC Adv.*, 2017, **7**, 290–299.
